# Exploring the Speciation of *trans*-Aconitic Acid in Aqueous Solution: Interactions with Divalent Metal Cations of Environmental Relevance

**DOI:** 10.1021/acsomega.6c01283

**Published:** 2026-05-04

**Authors:** Anna Irto, Rosita Cappai, Alessandro Amadeo, Massimiliano Peana, Giuseppe Cassone, Concetta De Stefano, Clemente Bretti

**Affiliations:** † Department of Chemical, Biological, Pharmaceutical and Environmental Sciences, 18980 University of Messina, Viale Ferdinando Stagno d’Alcontres, 31, Messina I-98166, Italy; ‡ Department of Chemical, Physical, Mathematical and Natural Sciences (DISCI), 9312 University of Sassari, Via Vienna 2, Sassari 07100, Italy; § Department of Chemistry, Biology and Biotechnologies, University of Perugia, Via dell’Elce di sotto, 8, Perugia 06123, Italy; ∥ Institute for Chemical-Physical Processes, National Research Council of Italy (CNR-IPCF), Viale Ferdinando Stagno d’Alcontres 37, Messina 98158, Italy

## Abstract

*Trans*-aconitic acid is a naturally occurring, biodegradable, unsaturated tricarboxylic acid that represents a promising sustainable alternative to conventional, poorly degradable chelating agents used in metal ion remediation. In this study, its acid–base behavior and chelating properties toward environmentally relevant divalent metal cations (Ca^2+^, Mg^2+^, Cu^2+^, Co^2+^, and Zn^2+^) were systematically investigated in an aqueous solution of potassium chloride (*I* = 0.10–1.00 mol dm^–3^) and at *T* = 298.15 K. A combined experimental and computational approach, including potentiometry, UV–vis and NMR spectroscopy, and Density Functional Theory (DFT) calculations, was employed to achieve a molecular-level thermodynamic characterization. The results indicate that *trans*-aconitic acid exhibits good sequestration efficiency, with a marked affinity toward Cu^2+^ and Mg^2+^ cations, as confirmed by pL_0.5_ and pM parameters as well as binding energy analyses. DFT calculations elucidated the microscopic acidities of the three carboxylic groups and rationalized the different interaction modes of Ca^2+^ and Mg^2+^. Overall, this study provides a comprehensive thermodynamic framework for *trans*-aconitic acid-metal interactions, supporting its potential application as an ecofriendly ligand for metal ion remediation and scale prevention in natural and industrial waters.

## Graphical abstract

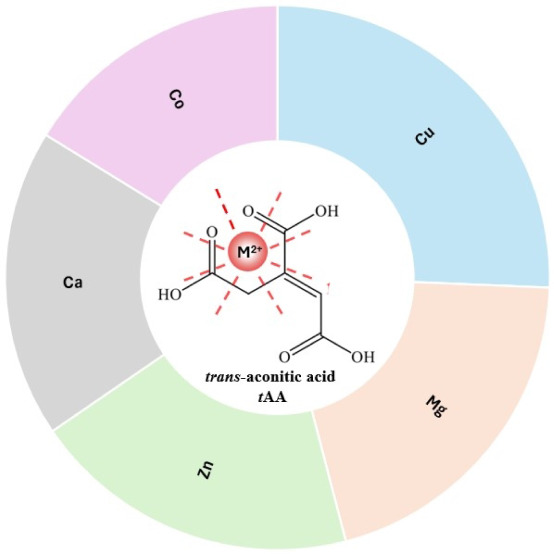

## 1 Introduction

Metal cations are ubiquitous in natural and anthropogenic systems and play an essential role in numerous biological, industrial, and environmental processes. However, elevated concentrations of both heavy and non-heavy metal cations in aquatic environments may arise from point and diffuse sources, including agricultural practices, industrial activities, and domestic waste, posing risks to ecosystems and human health.^1,2^ In this context, chelation represents a key mechanism for controlling the metal mobility, bioavailability, and toxicity in water and soil.

The development of effective, low-impact, and biodegradable chelating agents is therefore of growing interest, particularly as sustainable alternatives to conventional ligands such as EDTA, whose environmental persistence raises increasing concern.^3,4^ Organic poly-carboxylic acids of natural origin constitute a promising class of ligands for environmental metal sequestration.^5–7^ Among them, *trans*-aconitic acid (H_3_L, *t*AA; Scheme 1) is an unsaturated tricarboxylic acid widely occurring in nature, especially in sugarcane and sweet sorghum, where it represents the predominant six-carbon organic acid.^8,9^ Beyond its natural abundance, *t*AA can be obtained through industrial chemical synthesis from citric acid^10^ or via emerging biobased routes, including microbial production, which offer sustainable and potentially scalable alternatives.^11–13^ These features make *t*AA an attractive candidate for green chemistry applications that require biodegradable and low-toxicity ligands. From a structural standpoint, the presence of three carboxylic groups and a rigid unsaturated backbone suggests that *trans*-aconitic acid may exhibit versatile coordination modes and significant metal-binding ability in aqueous solution.

**Scheme 1:**
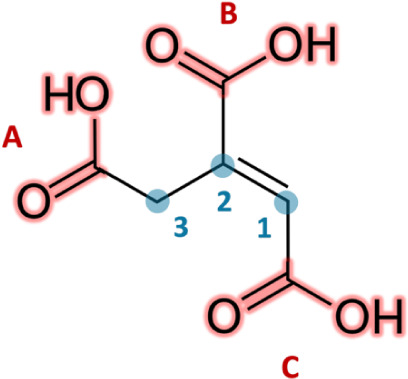
*Trans-*Aconitic Acid (*t*AA) Structure in the Fully Protonated Form (H_3_L)^a^

a The numbers stand for the ^1^H and ^13^C NMR titration peaks assignment, whereas capital letters serve for identifying specific sites for deprotonation and metal chelation (see along the text).

Indeed, *t*AA has been proposed for various industrial applications, including polymer cross-linking, plasticizers, metal–organic frameworks, and antiscalant formulations.^10,14–18^ Despite this potential, the fundamental thermodynamics and molecular features governing *t*AA-metal interactions in solution remain only partially understood. Previous studies have addressed the acid–base behavior of *trans*-aconitic acid and its interaction with alkali metal cations, as well as its sequestering ability toward selected environmentally relevant metal ions such as Cd^2+^, Mn^2+^, and Pb^2+^ under different experimental conditions.^19^ These investigations highlighted *t*AA as a competitive chelating agent when compared to other ligands. Additional studies have explored its antioxidant properties,^20^ its role in multicomponent solid formation,^21^ and its coordination behavior in crystalline metal–organic systems.^22^ However, comprehensive molecular-level and thermodynamic characterization of *t*AA interactions with other metal cations of environmental and biological relevance is still lacking. Proton affinity and metal-binding equilibria have not been jointly investigated using combined experimental and computational approaches yet. To fill this gap, the present work focuses on the complexation of *trans*-aconitic acid with Ca^2+^, Mg^2+^, Cu^2+^, Zn^2+^, and Co^2+^ in aqueous solution. These metal cations were selected to represent both alkaline earth and transition metals commonly encountered in natural waters, industrial systems, and environmental contamination scenarios, and are also relevant in processes such as scaling, nutrient cycling, and metal pollution. The study aims to elucidate the thermodynamic stability, speciation, and molecular features of *t*AA-metal complexes in KCl ionic medium over a range of ionic strengths (*I* = 0.10–1.00 mol dm^–3^) at *T* = 298.15 K. Experimental investigations based on potentiometry, UV–vis spectrophotometry, and ^1^H and ^13^C NMR spectroscopy are complemented by Density Functional Theory (DFT) calculations to provide a description of metal binding at both macroscopic and molecular levels. Ultimately, this integrated approach seeks to assess the potential of *trans*-aconitic acid as a biodegradable alternative to conventional chelating agents for applications such as metal remediation and antiscalant treatment in natural and industrial waters.

## 2 Methods

### 2.1 Materials, Procedures, and Apparatuses

All of the chemicals employed were obtained from Merck (Darmstadt, Germany) at the highest available purity and were utilized without additional purification. Further details are listed in Table S1. Various analytical methods and techniques, outlined briefly below, were used to carry out the studies; further details can be found in the Supporting Information and in Table S2. Potentiometric titrations were performed by means of a Metrohm (Herisau, Switzerland) 809 Titrando apparatus, consisting of an automatic buret and a combined glass electrode (Metrohm, model 6.0262.100). Spectrophotometric titrations were carried out utilizing a Varian (Agilent Scientific Instruments, California, U.S.A.) Cary 50 UV–vis spectrophotometer, equipped with an optical fiber probe. ^1^H and ^13^C NMR spectra were recorded in a 9:1 (v/v) H_2_O:D_2_O mixture, while two-dimensional ^1^H–^13^C heteronuclear multiple-bond correlation (HMBC) spectra were acquired in D_2_O, using a Bruker Ascend 400 MHz spectrometer (Bruker BioSpin, Billerica, MA, USA) equipped with a 5 mm automated tuning and matching broadband fluorine observation (BBFO) probe with *z*-axis gradients.

### 2.2 Programs and Calculations

The experimental data obtained from the various analytical techniques were processed by using appropriate computational tools. A description of the software employed is reported in the Supporting Information. Throughout the paper, uncertainties are given as 95% confidence intervals.

### 2.3 Density Functional Theory Calculations

All calculations were performed by means of the Gaussian 09 software.^23^ This latter, exploiting Density Functional Theory (DFT), enables the evaluation of the ground-state structures of selected molecular species. In this work, the B3LYP hybrid exchange and correlation functional, with 100% of exact exchange, was employed.^24–27^ Geometry optimizations of the molecular *trans*-aconitic (*t*AA) and tricarballylic (TA) acid structures were performed by employing the 6-311++G­(d,p) atomic basis set for all atoms. As for the simulation of the solvent, the continuum solvation model based on the quantum-mechanical charge density SMD was employed by setting typical parameters for water.^28^ After structural relaxation to the ground state, vibrational calculations were performed not only to establish the correctness of the previous calculations (i.e., absence of imaginary frequencies) but also to obtain the zero-point energy (ZPE) associated with each optimized molecular structure. In particular, the latter was included in those specific calculations from the frequency analysis of the vibrational modes. Nuclear quantum effects have to be taken into account carefully in proton transfer phenomena and proton affinity calculations in light of recent findings showing their relevance in water also at room temperature.^29^ Besides, ZPE values are essential for determining the proton affinity, which is defined as the negative of the change in enthalpy for the exemplary reaction H^+^ + B → H^+^B. With the aim of obtaining a complete scenario on the capabilities that *t*AA holds in donating protons, quantum-mechanical calculations were performed under implicit solvation and for different deprotonation states involving all the possible molecular deprotonation sites. This way, the proton affinity values were obtained by calculating the energy difference between the optimized neutral and deprotonated *t*AA molecules at the B3LYP/6-311++G­(d,p) level of theory using the following equation (eq 1):^30^

1 PA=−ΔH=−ΔE−ΔZPE+ΔEv(T)+C

The difference between the electronic energies is hence corrected by the difference between the ZPE (ΔZPE) of the species. The second and third terms in the latter equation are obtained from the frequencies of the normal modes of vibration by performing a vibrational analysis of the neutral and deprotonated species. The final term C introduces the correction for translational and rotational energy changes, assuming classical behavior, and a ΔnRT term is required to convert an energy to enthalpy, assuming an ideal gas behavior.

In addition to this series of calculations, simulations aimed at determining the binding energy associated with the sequestration of Ca^2+^, Mg^2+^, and Cu^2+^ by the *t*AA and the TA molecules were executed, both for the MH_2_L^+^ and the ML^–^ complexes. Of course, in the calculations involving the Cu^2+^ cation, due to the presence of 3*d* electrons, the ground state corresponded to the doublet spin state. This way, the most likely chelation sites were characterized at the molecular level. Although it might appear thermodynamically unlikely that *t*AA and TA form “naked” 1:1 complexes, the addition of a partial solvation structure involving a bunch of water/hydroxide species is suboptimal as well. To achieve a satisfactory description of the relaxation and stabilization processes taking place in more realistic descriptions of the liquid environment, *ab initio* molecular dynamics coupled with enhanced sampling techniques are in order so as to explicitly include fundamental entropic contributions and thermal fluctuations, whose specific investigation represents an interesting topic for future work.

### 2.4 Equilibria and Models for the Dependence on Ionic Strength

The data analysis permitted refining the values of equilibrium constants, expressed as follows:

2 $$j\;M^{2+}+i\;H^{+}+k\;L^{3-}=M_{j}H_{i}L_{k}^{(2j+i-3k)\;},\beta_{jik}$$

3 $$j\;M^{2+}+H_{i}L^{\left(i-3k\right)}=M_{j}H_{i}L_{k}^{(2j+i-3k)\;},K_{jik}$$

where M, H, and L represent the metal cation of interest, the proton, and *trans*-aconitate anion, respectively. When *k* = 0 and *i* < 0, eq 2 refers to metal cation hydrolysis; when *j* = 0, the equilibria are related to the overall (eq 2) and stepwise (eq 3) protonation constants of the ligand, generally denoted as β^H^_i_ and *K* ^H^_i_. The dependence of the stability constants on ionic strength was studied according to the variation of activity coefficients using the Debye–Hückel type equation reported below (eq 4). A deep and extensive discussion on this topic is provided in the Supporting Information of a previous work,^19^ and to avoid repetition, only the main equation used is reported here:

4 $${\log\beta }_{\mathrm{ijk}}={\log\beta }_{\mathrm{ijk}}^{0}-0.51\cdot z^{*}\cdot \frac{\sqrt{I}}{1+1.5\cdot \sqrt{I}}+C_{\mathrm{ijk}}{\mathrm{\cdot I}}_{c}$$

where logβ and logβ^0^ are the equilibrium (protonation, stability, or formation) constants at a given ionic strength and at infinite dilution, respectively; z* = Σ­(charge)^2^_reactants_ – Σ­(charge)^2^_products_, and *C* is an empirical parameter that accounts for the ionic strength dependence.

### 2.5 Metal Sequestration Affinity

The sequestering ability of *trans*-aconitic acid toward Ca^2+^, Mg^2+^, Cu^2+^, Zn^2+^, and Co^2+^ was assessed by determining the pL_0.5_ ^31^ under various conditions such as *T* = 298.15 K, different ionic strengths, and pH values in a KCl ionic medium, as well as under the average seawater conditions (*I* = 0.72 mol dm^–3^ and pH ∼ 8.2).^32^ The pL_0.5_ is a quantitative empirical parameter and indicates the total ligand concentration needed for the sequestration of 50% of trace concentration of metal cation in solution (*c*_M_ ∼ 10^–9^ mmol dm^–3^). Other details can be found in ref 33. The higher the pL_0.5_, the greater the sequestering ability. This parameter may be expressed by means of the following sigmoidal-type Boltzmann equation (eq 5), whose graphical representation is a dose–response curve with asymptotes approaching 0 as pL → +∞ and 1 as pL → −∞:

5 $$\chi_{M}=\frac{1}{{1+10}^{{(\mathrm{pL}-\mathrm{pL}}_{0.5})}}$$

where χ_M_ is the molar fraction of metal cation sequestered by the ligand, pL = −log*c*_L_ and pL_0.5_ = −log*c*_L_ at χ_M_ = 0.5. Although pL_0.5_ may numerically coincide with parameters such as BC_50_ ^34^ or Schwarzenbach’s^35^ apparent formation constant under specific conditions, it offers a more intuitive scale for assessing the sequestering strength of a ligand. Its main advantage lies in its practical determination: pL_0.5_ can be obtained using standard speciation tools without requiring detailed expertise in complex equilibrium or mass-balance modeling. The evaluation of the sequestering ability could find concrete application in processes involving the usage of a chelating agent, such as for the remediation of contaminated sites or the treatment of polluted waters, trying to optimize the working conditions.

Furthermore, the ligand affinity or efficacy toward the divalent metal cation under study was evaluated by determining the pM^36^ parameter (eq 6) at *c*_M_ = 0.001 mmol dm^–3^, *c*_L_ = 0.010 mmol dm^–3^, and typical ionic strength and pH conditions of marine waters (e.g., *I* = 0.72 mol dm^–3^ and pH ∼ 8.1):^32^

6 $$pM=-log\;{[M]}_{free}=-log\;{[metal\;cation]}_{free}$$

The higher the free metal concentration ([M]_free_) is, the lower the M^2+^/ligand affinity will be, leading to a lower pM value and a subsequent minor “strength” of interactions.

## 3 Results and Discussion

### 3.1 Ligand Acid–Base Properties

The *trans*-aconitic acid (*t*AA) structure (Scheme 1) displays three potential protonable carboxylic groups, determinable at pH ∼ 2.5, 3.8, and 5.4, respectively. The ligand protonation constants were previously published^19^ by this research group by means of potentiometry at various ionic strengths (*I* = 0.10–1.00 mol dm^–3^) in sodium chloride, potassium chloride, and tetraethylammonium iodide supporting electrolytes, and at different temperatures (*T* = 288.15–310.15 K). These data were successfully compared with the ones reported by Berto *et al*.^37^ and Kostakis *et al*.^38^ at the same temperature and *I* = 0.10 mol dm^–3^ in KCl_(aq)_ and KNO_3(aq)_.

In the present paper, the *t*AA acid–base properties were investigated by UV–vis spectrophotometry as well as ^1^H and ^13^C NMR spectroscopy at *I* = 0.15 mol dm^–3^ in KCl_(aq)_ and *T* = 298.15 K. Data analysis performed using the above-mentioned computer programs allowed the determination of three protonation constants, reported in Table 1, which are in quite good accordance with the literature values under similar experimental conditions.

**Table 1:** Experimental and Literature Overall^a^ and Stepwise^b^ Ligand Protonation Constants at Various Ionic Strengths and *T* = 298.15 K

Analytical technique	*I*/mol dm^–3^	Ionic medium	log*K* ^H^_1_ ^b^	logβ^H^_2_ ^a^ (log*K* ^H^_2_)^b^	logβ^H^_3_ ^a^ (log *K* ^H^_3_)^b^	Ref.
Potentiometry	0.10	KCl	5.611	9.57 (3.959)	12.334 (2.764)	^19^
Potentiometry/UV–vis	0.10	KCl	5.56	9.50 (3.94)	12.24 (2.74)	^37^
Potentiometry	0.10	KNO_3_	5.61	9.56 (3.95)	12.28 (2.72)	^38^
UV–vis	0.15	KCl	5.67 ± 0.02^c^	9.46 ± 0.02 (3.79)	12.29 ± 0.05 (2.83)	This study
^1^H NMR	0.15	KCl	5.64 ± 0.05^c^	9.62 ± 0.07 (3.98)	12.34 ± 0.07 (2.72)	This study
^13^C NMR	0.15	KCl	5.49 ± 0.02^c^	9.46 ± 0.04 (3.96)	12.16 ± 0.06 (2.71)	This study

a logβ^H^_i_ related to equilibrium in eq 2.

b log*K* ^H^_i_ referred to equilibrium in eq 3.

c ± std. dev.

Figure S1a shows the spectrophotometric ligand spectra collected at different pH values. Since no significant absorbance was observed in the Visible region for any measurements, the spectrum is only shown up to λ = 300 nm for graphical clarity. An absorption band with λ_max_ = 217 nm can be noticed at pH ∼ 2.0, which decreases its intensity at pH ≥ 2.5, accompanied by a subsequent hypsochromic shift at pH ≥ 3.0. Then, the signal starts to lose its resolved shape at pH ∼ 4.1 and, more significantly, at pH ∼ 5.8, with the absorbance rising up to the final pH (pH ∼ 10.5). Once the *t*AA protonation constants were determined, the HypSpec program^39^ also allowed the UV–vis data deconvolution and, therefore, the calculation of the molar absorptivity (ε/mol^–1^ dm^3^ cm^–1^) values for each protonated and deprotonated *trans*-aconitic acid species, graphically represented in Figure S1b.

The ligand ^1^H NMR spectra (Figure S2) recorded over the pH range 1.93–10.17 exhibited a single set of pH-dependent average resonances, suggesting that a fast mutual exchange occurs on the NMR time scale, despite the involvement of multiple species in the protonation equilibria. Proton groups 1 and 3 (Scheme 1) exhibit a progressive upfield shift as the pH increases, consistent with deprotonation-induced shielding effects. In this light, each recorded chemical shift was considered correspondent, at the various pH of analysis, to the mol-fraction weighted average of the single δ/ppm values for the species taking part in the ligand protonation equilibria. In addition, for supporting the calculation of the previously determined protonation constants, the HypNMR software^40^ allowed the refinement of the individual chemical shifts for the L^3–^, HL^2–^, H_2_L^–^, and H_3_L^0^_(aq)_ species (Table S3), afterward employed as input for the M^2+^/*t*AA NMR data analysis. The acid–base calculation reliability was also confirmed by the large overlap, shown in Figure 1, between the experimental (observed) and calculated chemical shifts for both types of ^1^H NMR-active nuclei present in the ligand structure (Scheme 1).

**Figure 1:**
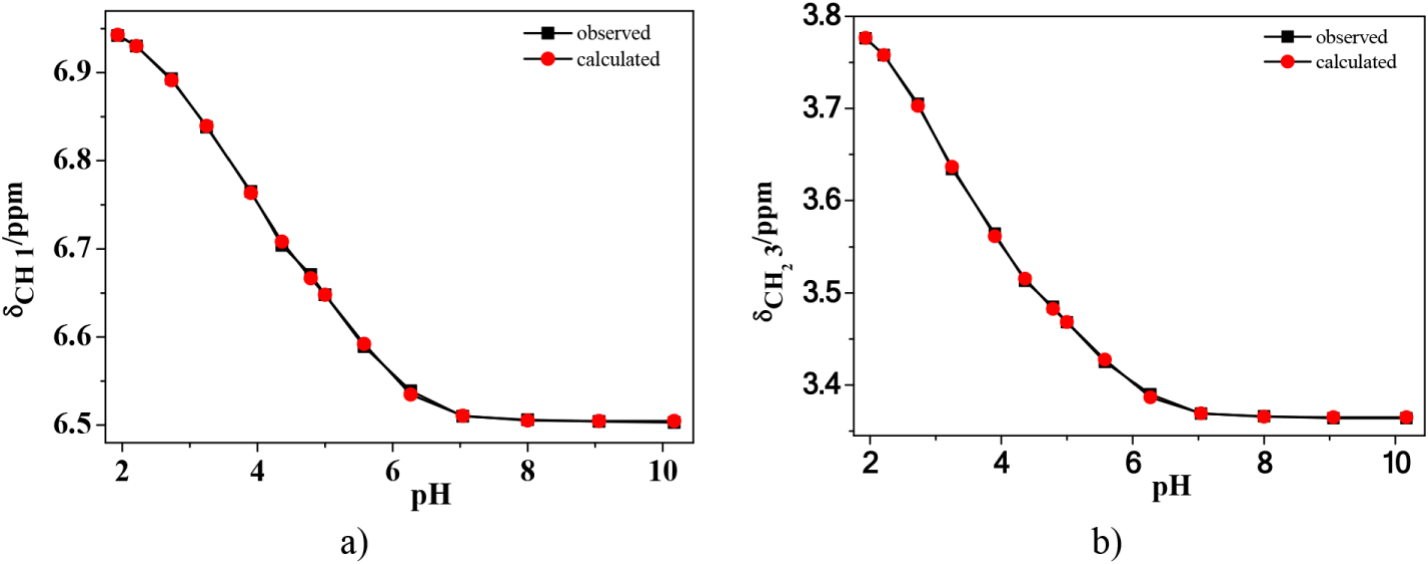
Observed (■-black) and calculated (•-red) values of proton chemical shifts of a) 1 and b) 3 nuclei of *trans*-aconitic acid *vs* pH at *c*_L_ = 5.00 mmol dm^–3^, *I* = 0.15 mol dm^–3^ in KCl_(aq)_ and *T* = 298.15 K.

The acid–base behavior of *trans*-aconitic acid was investigated by one-dimensional ^13^C NMR titration over a wide pH range (∼1.9–7.3) at *c*_L_ = 100 mmol dm^–3^ (Figure 2, Figure S3 and Table S4). The pH-dependent chemical shifts (δ) provide direct insight into the stepwise deprotonation of the three carboxylic groups A, B, and C. All three carbons exhibit progressive downfield shifts with increasing pH, consistent with successive deprotonation and increased electron withdrawal upon formation of carboxylate species.

**Figure 2:**
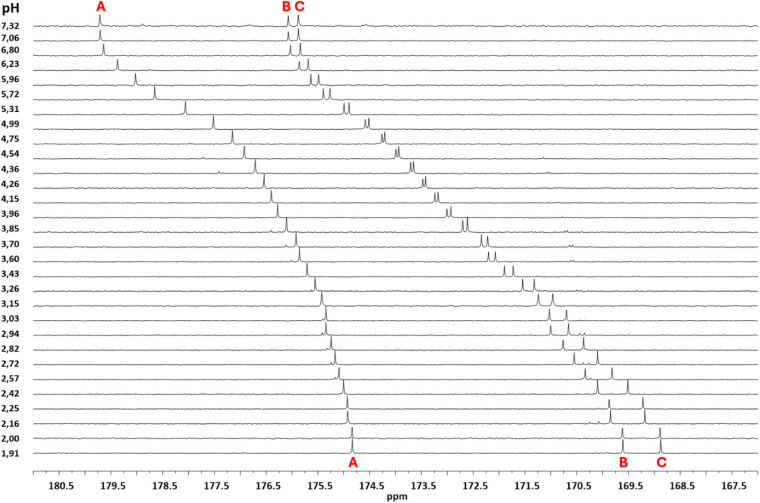
^13^C NMR spectra of *trans*-aconitic acid recorded in a 9:1 (v/v) H_2_O:D_2_O mixture over the pH range 1.91–7.32 at *c*_L_ = 100 mmol dm^–3^, *I* = 0.15 mol dm^–3^ in KCl_(aq)_ and *T* = 298.15 K.

The overall chemical shift changes (Δδ) differ markedly: carbon in C displays the largest variation (∼7 ppm), followed by carbon in B (∼6.5 ppm), whereas carbon in A varies less (∼4.9 ppm). This trend reflects the different electronic environments of the carboxyl groups, with conjugation to the C=C bond enhancing the sensitivity of carbons in B and C compared to the nonconjugated terminal group A.

To quantitatively model the pH dependence, a global triprotic Henderson–Hasselbalch-type model (more details in Supporting Information) was applied simultaneously to all carbon signals (see Figure S3–NMR), including the carboxyl carbons (A, B, C), the olefinic carbons (1 and 2), and the aliphatic methylene carbon 3. Each observed chemical shift was expressed as a population-weighted average over four microscopic protonation states, with the three macroscopic p*K*_a_ values constrained to be global parameters shared across all carbons. The model reproduces the experimental δ *vs* pH curves with high fidelity, yielding p*K*_a1_ = 2.72, p*K*_a2_ = 3.97, and p*K*_a3_ = 5.49 (log*K* ^H^_1_ = 5.49, log*K* ^H^_2_ = 3.97, log*K* ^H^_3_ = 2.72, Table 1).

Numerical derivatives of δ with respect to pH (dδ/dpH), calculated directly from the experimental data, were compared to analytical derivatives obtained from the fitted triprotic model (Figure S4). Excellent agreement was observed in both the magnitude and position of the maxima, confirming the robustness of the model. Minor discrepancies at the pH extremes are attributed to experimental noise and discrete pH sampling. Analysis of the derivatives identified the site-specific sequence of deprotonation. Carbon in C exhibits the earliest and most pronounced maximum in dδ/dpH, indicating that the terminal conjugated carboxyl group C deprotonates first. This assignment is corroborated by the early and intense derivative response of carbon 1, directly bonded to C. The second deprotonation primarily involves carbon B, with a derivative maximum at slightly higher pH, mirrored by carbon 3, directly conjugated to B. Finally, carbon in A shows the latest and broadest derivative maximum, consistent with the final deprotonation step; this is echoed by the aliphatic carbon 3 bonded to A. Collectively, the concerted behavior of the carboxyl carbons and their neighboring “satellite” carbons establishes the deprotonation sequence as C → B → A, reflecting both conjugation effects and local electronic environments.

Complementary computational simulations at the hybrid DFT level were performed to assess the intrinsic acidity of the three carboxyl sites. Geometry optimizations and proton affinity calculations were carried out at the B3LYP/6-311++G­(d,p) level with implicit aqueous solvation. The calculated proton affinities for the reaction H_2_L^–^ + H^+^ → H_3_L for sites B, C, and A are reported in Table 2. Since proton affinities are inversely related to acidity, the corresponding deprotonation reaction (H_3_L → H_2_L^–^ + H^+^) indicates that the two conjugated carboxyl groups (B and C) are intrinsically more acidic than the nonconjugated terminal group A. The slight difference in intrinsic acidity between B and C lies within a range readily modulated by solvation and intramolecular electrostatic effects, rendering B and C effectively quasi-degenerate. On the other hand, it has to be noticed that, according to our calculations, also the proton affinity difference between the C and A sites lies in ∼3 kcal mol^–1^.

**Table 2:** Proton Affinity of the Different Sites in *Trans-*Aconitic Acid for the Reaction H_2_L^–^ + H^+^ → H_3_L Determined from DFT Calculations at the B3LYP/6-311++G­(d,p) Level

Carboxylic Site	Proton Affinity (kcal mol^–1^)
A	277.60
C	274.76
B	271.25

This way, while our computational results are qualitatively consistent with the ^13^C NMR titration data, which is capable of probing macroscopic equilibria in solution, the lack in our modeling of explicit solvation dynamics might be the source of the recorded quantitative discrepancy between simple static DFT calculations and the experimental evidence. In fact, the strongest spectroscopic response at the first deprotonation step corresponds to carbon in C, confirming that the experimentally observed sequence C, B, A reflects a combination of intrinsic acidity and environmental effects, including solvation and charge–charge interactions. The inclusion of carbons 1, 2, and 3 provides further validation, as their δ­(pH) variations propagate consistently with the corresponding carboxyl deprotonation events.

### 3.2 Interaction of *trans*-Aconitic Acid with M^2+^

The interactions of *trans*-aconitic acid with Ca^2+^, Mg^2+^, Cu^2+^, Co^2+^, and Zn^2+^ were studied, at first, by means of potentiometric titrations in KCl_(aq)_ at different ionic strengths and *T* = 298.15 K. The experiments were performed in the pH ranges 2.0 ≤ pH ≤ 10.0 for Ca^2+^ and Mg^2+^systems, 2.0 ≤ pH ≤ 8.0 for the Zn^2+^ one, and up to pH ∼ 6.0 for the Co^2+^ and Cu^2+^/L^3–^ systems, owing to the formation of sparingly soluble species that prevented the investigation at more alkaline pH values. The formation of metal hydrolytic species (e.g., M_j_(OH)_i_^(2j–i)^, taken from refs. 41 and 42) as well as the ligand acid–base behavior in KCl ionic medium were considered and kept fixed during the calculations (Table S5). Different speciation models were tested, and the most suitable ones were selected based on typical selection criteria,^43^ also ensuring consistency with literature data reported for similar ligands.^44^ The most reliable speciation schemes for M^2+^/L^3–^ systems include three main species, namely MH_2_L^+^, MHL^0^_(aq)_, and ML^–^ for Ca^2+^, Mg^2+^, and Zn^2+^; the ML^–^ complex for Cu^2+^; and the two ML and MHL^0^_(aq)_ species for Co^2+^. In the case of copper­(II)-containing system, the possible formation of CuH_2_L^+^, CuHL^0^_(aq)_, and CuL­(OH)^2–^ was also checked; however, although these species were sometimes detected, their formation did not reach significant formation percentages (<5%), and their inclusion did not improve the fit quality. Similar considerations can be made for all the systems concerning the possible determination of M_2_L^+^ or ML_2_^4–^ species, always consistently rejected by the BSTAC4 computer program^45^ or reached very low percentages (1–2%).

In Table S6, in addition to the potentiometric formation constants of the M^2+^/*t*AA systems at *I* = 0.15 mol dm^–3^ in KCl_(aq)_, the maximum formation percentages (max (%)) and pHs (pH_max_) of each species are shown. As noticeable in Tables 3 and S6–S7, the stability of the metal–ligand species is quite low and similar among the various metal cations. To better illustrate the distribution of the M^2+^/*t*AA species through the investigated pH ranges, the respective speciation diagrams were drawn for each system, and the resulting graphs are reported in Figures 3 and S5. In the case of Ca^2+^ and Mg^2+^ systems (Figure 2a), at *I* = 0.15 mol dm^–3^ a quite similar pH trend was observed for the two metal cations; however, slightly higher percentages were found for the Mg^2+^/L^3–^ species with respect to the Ca^2+^ ones, as also highlighted in Table S6.

**Table 3:** Formation Constants of *Trans*-Aconitic Acid with Metal Cations and Ionic Strength Dependence Parameters (Valid in KCl_(aq)_) in the Molar Concentration Scale at Infinite Dilution, *T* = 298.15 K

Species	Equilibrium	z*	p*	$$\log\beta_{ijk}^{0}$$	$\log K_{ijk}^{0}$ ^a^	*C*_ijk_
CaL^–^	Ca^2+^ + L^3–^ = CaL^–^	12	1	4.34 ± 0.01^b^	4.34	0.27 ± 0.02^b^
CaHL^0^_(aq)_	Ca^2+^ + H^+^ + L^3–^ = CaHL^0^_(aq)_	14	2	9.55 ± 0.02	3.23	0.38 ± 0.02
CaH_2_L^+^	Ca^2+^ + 2 H^+^ + L^3–^ = CaH_2_L^+^	14	3	13.18 ± 0.01	2.46	0.47 ± 0.01
Mg L^–^	Mg^2+^ + L^3–^ = Mg L^–^	12	1	4.36 ± 0.02	4.36	0.35 ± 0.03
MgHL^0^_(aq)_	Mg^2+^ + H^+^ + L^3–^ = MgHL^0^_(aq)_	14	2	9.79 ± 0.02	3.47	0.44 ± 0.03
MgH_2_L^+^	Mg^2+^ + 2 H^+^ + L^3–^ = MgH_2_L^+^	14	3	13.64 ± 0.02	2.92	0.47 ± 0.03
ZnL^–^	Zn^2+^ + L^3–^ = ZnL^–^	12	1	4.45 ± 0.03	4.45	0.96 ± 0.06
ZnHL^0^_(aq)_	Zn^2+^ + H^+^ + L^3–^ = ZnHL^0^_(aq)_	14	2	9.88 ± 0.02	3.56	1.11 ± 0.04
ZnH_2_L^+^	Zn^2+^ + 2 H^+^ + L^3–^ = ZnH_2_L^+^	14	3	13.67 ± 0.02	2.95	0.66 ± 0.03
CuL^–^	Cu^2+^ + L^3–^ = CuL^–^	12	1	4.34 ± 0.03	4.34	0.32 ± 0.06
CoL^–^	Co^2+^ + L^3–^ = CoL^–^	12	1	4.07 ± 0.02	4.07	1.13 ± 0.02
CoHL^0^_(aq)_	Co^2+^ + H^+^ + L^3–^ = CoHL^0^_(aq)_	14	2	9.61± 0.02	3.29	0.96 ± 0.04

a log *K* ^0^_ijk_ refers to eq 3.

b ± std. dev.

**Figure 3:**
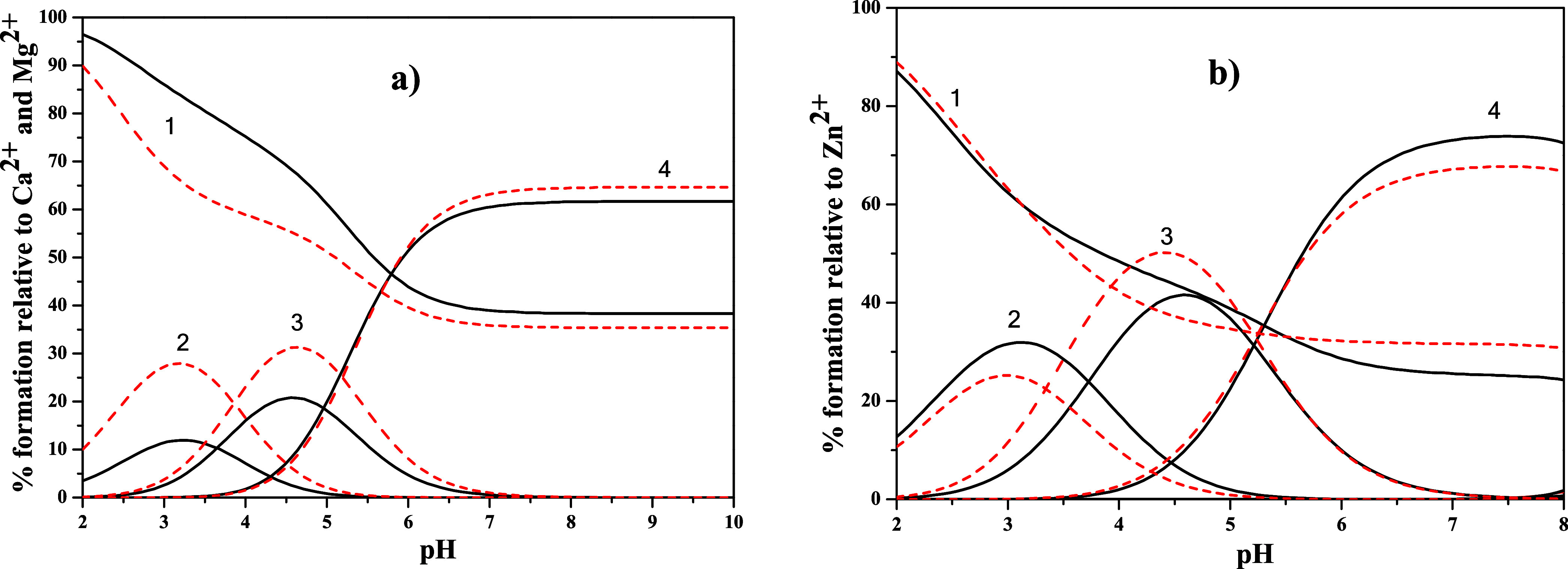
Speciation diagrams of: a) Ca^2+^/L^3–^ (solid line) and Mg^2+^/L^3–^ (dashed line) complexes at *I* = 0.15 mol dm^–3^, *T* = 298.15 K; b) Zn^2+^/L^3–^ species at *I* = 0.15 mol dm^–3^ (solid line) and *I* = 1.00 mol dm^–3^ (dashed line), *T* = 298.15 K. Analytical concentrations: *c*_M_ = 0.001 mol dm^–3^; *c*_L_ = 0.003 mol dm^–3^. Species: 1 free M^2+^, 2 MH_2_L^+^, 3 MHL^0^, and 4 ML^–^.

As detailed in Section 2, a series of DFT calculations was performed to investigate the interaction between the divalent cations such as Ca^2+^ and Mg^2+^ with the *trans*-aconitate ligand to form the MH_2_L^+^ and ML^–^ complexes while water was treated as a continuous dielectric medium. Although Ca^2+^ and Mg^2+^ behave similarly to each other, as shown in Figure 3a, DFT computations show that Ca^2+^ favorably binds at site B (Scheme 1), while the most likely site for the binding of Mg^2+^ to form the MgL^+^ species is between the two carboxylate groups A and B. At the roots of such a different behavior there might be the bigger ionic radius of Ca^2+^ (1.06 Å) with respect to that of Mg^2+^ (0.72 Å), as reported by Shannon.^46^ This feature could explain the experimentally observed discrepancies reported in Figure 3a.

In Figure 3b, the Zn^2+^/*t*AA distribution was compared at different ionic strengths, observing that the speciation is not strongly affected by the increase in this variable. Nevertheless, the complexation of the protonated species is shifted to more alkaline pH values (i.e., ∼0.2 pH units). Accordingly, the percentages of 30%, 40%, and 70%, also listed in Table S6, observed at pH ∼ 3.2, 4.6, and 6.5 for ZnH_2_L^+^, ZnHL^0^_(aq)_, and ZnL^–^ species, respectively, at lower ionic strength, can be compared with the values of 25%, 50%, and 65% detectable at similar pHs for the same complexes at *I* = 1.00 mol dm^–3^. Analogously, a slight ionic strength effect can also be noticed in the formation constants and pH in the case of the Co^2+^/ligand (Figure S5a) species. As for the system containing Cu^2+^ (Figure S5b, Table S6), the CuL^–^ species achieves 60% at pH ∼ 6.0, where the hydrolysis of Cu_2_(OH)_2_^2+^ also starts.

In the case of Ca^2+^, Mg^2+^, and Zn^2+^/L^3–^ systems, to confirm the accuracy and robustness of the potentiometric findings, ^1^H NMR measurements were performed at *I* = 0.15 mol dm^–3^ in the pH range ∼ 2.0–11.0. The spectra (Figures S6–S8) recorded in the 9:1 (v/v) H_2_O:D_2_O mixture exhibited single sets of pH-dependent averaged resonances. This observation is consistent with previous findings for *trans*-aconitic acid protonation, where a similar behavior was noted. Despite the involvement of multiple species in the protonation equilibria, the recorded spectra indicate the presence of a fast exchange regime on the NMR time scale, leading to coalescence into single averaged signals. This suggests that ligand coordination and protonation processes occur in rapid equilibrium, preventing the resolution of distinct resonances for individual species.

For the various systems, the HypNMR software^40^ allowed the successful determination of stability constants (Table S8) in good accordance with the potentiometric data (Table S6) and the calculation of the individual chemical shifts (Table S4) for each Ca^2+^, Mg^2+^, and Zn^2+^ complex species. Moreover, the accuracy of these calculations was validated by the close agreement between the observed and calculated chemical shifts for the two ^1^H NMR active nuclei (Scheme 1). An example of this satisfactory matching is shown in Figure S9 for the Zn^2+^/L^3–^ system.

In addition, in the case of Mg^2+^/*t*AA systems, ^13^C NMR measurements were performed at *c*_L_ = 100 mmol dm^–3^ in the pH range ∼ 1.8–7.3. In particular, chemical shift variations (Δδ, parts per million) for carbon in A, B, and C were determined as a function of pH in the presence of Mg^2+^. To visualize the pH-dependent behavior of the different sites and facilitate comparison, two complementary heatmap representations were generated: (i) a continuous heatmap of the absolute Δδ values (Figure S10) and (ii) a normalized heatmap highlighting competition between binding sites (Figure 4).

**Figure 4:**
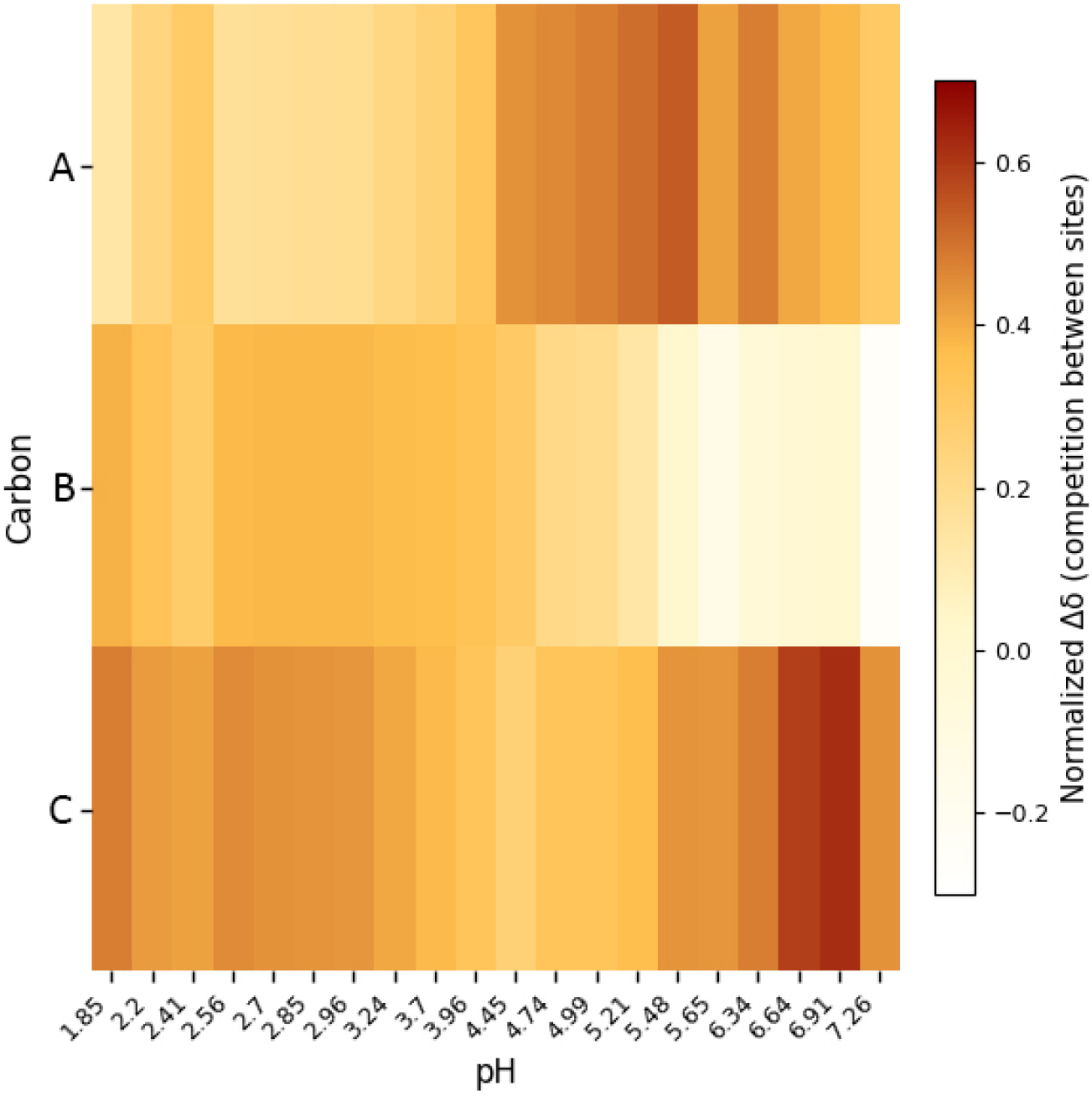
Heatmap of normalized chemical shift variations (Δδ_norm_) defined as ${\Delta \delta }_{\mathrm{i norm}}=\frac{{\Delta \delta }_{i}}{\left|{\Delta \delta }_{A}\right|+\left|{\Delta \delta }_{C}\right|+\left|{\Delta \delta }_{D}\right|}$ highlighting competition between binding sites A, B, and C as a function of pH. Normalization was performed for each pH value by scaling Δδ values to the sum of their absolute contributions.

The absolute Δδ heatmap reveals the overall sensitivity of each site to pH variations. At acidic pH, C exhibits the largest chemical shift changes, indicating a dominant interaction or stronger environmental perturbation. With increasing pH, carbon in A progressively shows larger Δδ values, particularly above pH ∼ 4.5, suggesting increased involvement in Mg^2+^ coordination or structural rearrangements, while B displays more moderate shifts and becomes negative at higher pH, indicating a reduced contribution. Although the absolute Δδ heatmap effectively captures the magnitude of chemical shift variations, it does not directly reflect site competition. This information is provided by the normalized heatmap, which highlights the redistribution of relative site contributions with pH. In this representation, site C dominates at low pH, site A becomes increasingly competitive at intermediate and high pH, and site B progressively loses relative weight. The ^13^C experimental data were also elaborated using the HypNMR software,^40^ obtaining values in quite good agreement with the ones refined using the other analytical techniques (Tables S6, S8).

In addition, UV–vis spectrophotometric titrations were carried out for all the investigated metal-to-ligand interactions at *I* = 0.15 mol dm^–3^ over the pH interval ∼ 2.0–10.5. Unfortunately, only the formation of some species, such as the CaL^–^, MgL^–^, ZnHL^0^_(aq)_, ZnH_2_L^+^, and CuL^–^, was feasible by employing HypSpec.^40^ The determined stability constants are log *K*_101_ = 2.76 ± 0.35, 2.86 ± 0.09, and 3.0 ± 0.7 for Ca^2+^, Mg^2+^, and Cu^2+^, respectively. In the case of Zn^2+^, logβ_111_ = 8.96 ± 0.16 (log*K*_111_ = 3.29) and logβ_121_ = 12.03 ± 0.05 (log*K*_121_ = 2.57) were refined. These values are in quite good agreement among the various analytical techniques employed to perform the solution studies. Nevertheless, owing to the low formation percentages of the mentioned species, possibly due to the much smaller component concentrations employed by spectrophotometry with respect to potentiometry and ^1^H NMR (i.e., 0.01–0.05 mmol dm^–3^ *vs* 1.0–7.0 and 1.6–5.0 mmol dm^–3^, respectively), as well as the higher errors in the statistical parameters and the very low or not significant calculated molar absorptivity (ε/mol^–1^ dm^3^ cm^–1^) values, the obtained UV–vis data were not considered accurate by the authors.

DFT-based calculations of the binding energy between L^3–^ (the most abundant form of *trans-*aconitic acid at pH ∼ 8.1) and Ca^2+^, Mg^2+^, and Cu^2+^ to form the ML^–^ species could possibly explain the reason why L^3–^ better sequestrates Cu^2+^ and Mg^2+^ metal cations with respect to Ca^2+^, as indicated in Figure 3. In fact, the binding energy for CaL^–^ is only −15.67 kcal mol^–1^, while those for MgL^–1^ and CuL^–^ are −59.75 and −55.19 kcal mol^–1^, respectively. Although these values testify to the stronger interactions established by *t*AA with Mg^2+^ and Cu^2+^, other factors might be in play to explain the (slightly) higher sequestration capability of *t*AA toward Cu^2+^ with respect to that featuring the MgL^–^ behavior (e.g., explicit dynamical role of the solvent, thermodynamic fluctuations, etc.). As a reference, the structures of the complexes optimized at the B3LYP/6-311++G­(d,p) level are reported in Figure 5, where the most thermodynamically favored site for binding is between the A and B sites for all systems (Scheme 1).

**Figure 5:**
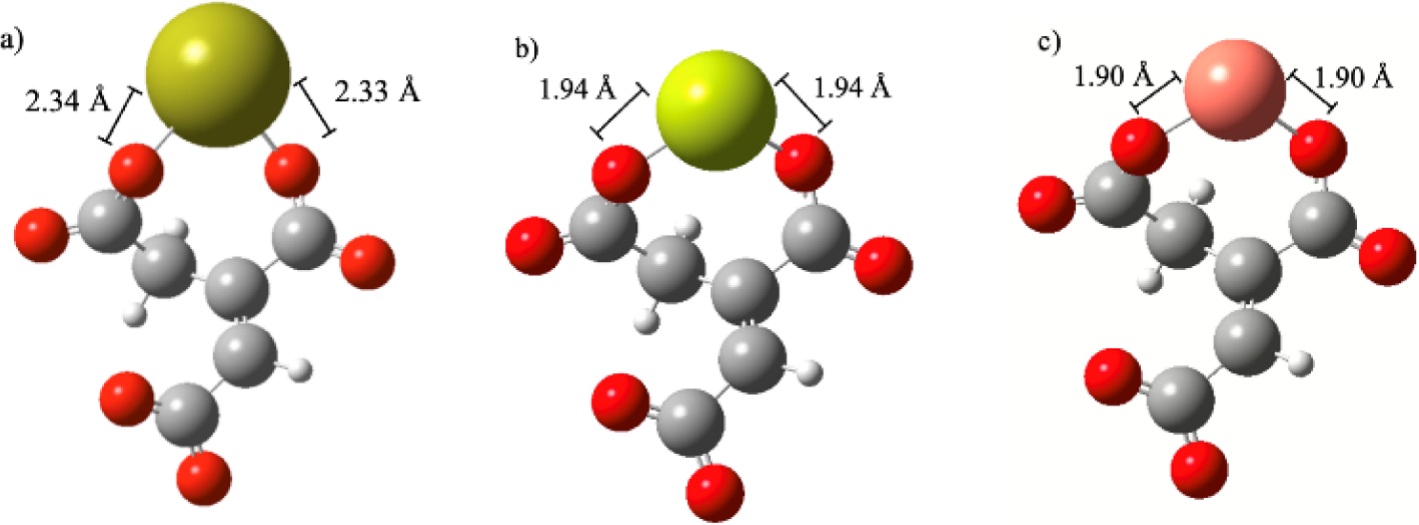
Molecular geometries optimized at the B3LYP/6-311++G­(d,p) level under implicit water solvation conditions of *trans*-aconitic acid chelating a) Ca^2+^, b) Mg^2+^, and c) Cu^2+^ in the ML^–^ complex.

### 3.3 Sequestering Ability and Metal Affinity

The sequestering ability of *trans*-aconitic acid toward the five divalent metal cations was assessed by determining the pL_0.5_ ^31^ at various ionic strengths in KCl supporting electrolyte and different pHs. The sequestration data are reported in Table S9. To mention only some examples, as noticeable in Figure S11, in the case of Ca^2+^/L^3–^ system at *I* = 0.15 mol dm^–3^, the pL_0.5_ increases up to pH ∼ 7.4, then remains constant until the final pH (∼10.0), possibly due to the very similar speciation in the pH interval 7.4–10.0, where the CaL^–^ is the only complex present in solution at significant and unchanged formation percentages.

Owing to the relatively weak Zn^2+^ and Cu^2+^/ligand complexes (Tables 3, S6–S8), the already previously mentioned observed precipitation could be likely attributable to the formation of sparingly soluble Zn­(OH)_2(s)_, ZnO_(s)_, Cu­(OH)_2(s)_, and CuO_(s)_ species,^42^ which restricts the pL_0.5_ calculation to a narrow pH range. Nevertheless, the studies reported by Powell *et al*.^47^ on the copper­(II) and zinc­(II) speciation in aqueous solutions at *c*_M_ = 10^–6^ mmol dm^–3^, and in the presence of high concentrations of chloride, carbonate, sulfate, and other anions typical of seawater composition,^32^ suggest that the interactions with these anions prevent the precipitation, enabling the pL_0.5_ calculation up to pH ∼ 8.1. As regards Co^2+^, as previously reported for Cu^2+^ and Zn^2+^, and by considering the real metal concentration in the same environmental matrix,^48^ the ligand sequestering ability was also evaluated up to seawater pH: the pL_0.5_ rises as the variable increases. Concerning the ionic strength effect, for the various M^2+^ the sequestration decreases as the *I*/mol dm^–3^ rises, with, in some cases, a tendency inversion occurring at *I* = 1.00 mol dm^–3^, reflecting the behavior of some stability constants.

Furthermore, the M^2+^ sequestration by the ligand was likened under pH and ionic strength conditions typical of marine water, such as pH ∼ 8.1 and *I* = 0.72 mol dm^–3^. For all the systems, prior to carry out the pL_0.5_ calculations under these conditions, the stability constants were calculated at the aforementioned ionic strength using eq 4. The ligand displayed the pL_0.5_ trend shown in Figure 6 and Table S9, suggesting that Cu^2+^ can be more successfully sequestered by the ligand with respect to the other divalent metal cations:

$$Cu^{2+}\left(3.71\right)>Mg^{2+}\left(2.96\right)>Zn^{2+}\left(2.83\right)>Ca^{2+}\left(2.66\right)>Co^{2+}\left(2.35\right)$$

**Figure 6:**
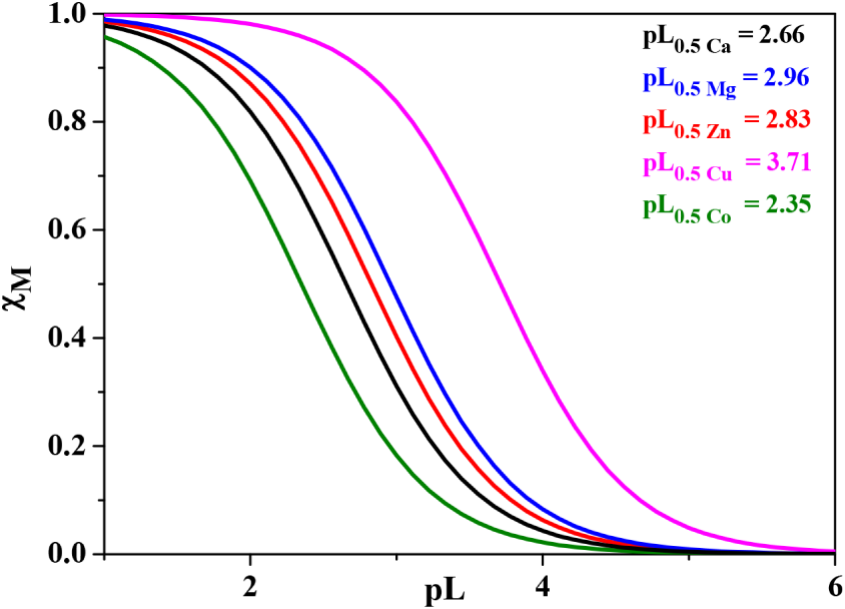
*Trans*-aconitic acid sequestration diagrams toward Ca^2+^, Mg^2+^, Zn^2+^, Cu^2+^, and Co^2+^ at pH ∼ 8.1 at *I* = 0.72 mol dm^–3^ in KCl_(aq)_ and *T* = 298.15 K.

In addition, the effectiveness of *trans*-aconitic acid toward the metal cation under study was assessed by calculating the pM values^36^ at the above-mentioned seawater ionic strength and pH. Consistent with the pL_0.5_ findings, the ligand shows a stronger, although not so markedly high, affinity for Cu^2+^ compared to the other M^2+^ investigated in this paper. The calculated pM values are

pCu(6.32)>pZn(6.06)∼pMg(6.01)∼pCo(6.01)∼pCa(6.00)

### 3.4 Literature Comparisons

As already pointed out,^19^ thermodynamic data in the literature regarding *trans*-aconitic interaction toward metal cations are very scarce, and only a few of them meet the criteria for critical selection. In fact, in the NIST^44^ and JESS^49^ databases, only data for Ca^2+^, Sr^2+^, and Mn^2+^ are reported.^50,51^ Values of log*K*_110_ = 1.50, 1.51, and 2.27 were determined, respectively, by equilibrium exchange technique at pH ∼ 7.2–7.3, *I* = 0.16 mol dm^–3^, and *T* = 298.15 K, in veronal buffer prepared with both sodium diethyl barbiturate and sodium acetate solutions (9.52 mmol dm^–3^).^50,51^ These conditions are different with respect to the ones reported in this work; therefore, making comparisons does not represent an easy task. Some solid-state studies demonstrated that *t*AA in the presence of metal cations such as Cu^2+^, Zn^2+^, and Cd^2+^ can form various (e.g., distorted octahedral, square tetrahedral) geometries.^52–54^ To the best of the authors’ knowledge, no data were published in the literature regarding the *cis*-aconitic acid interaction with Ca^2+^, Mg^2+^, Cu^2+^, Co^2+^, and Zn^2+^.

Some comparisons can be made using tricarballylic acid (TA, Figure S12), which resembles *trans*-aconitic in structure, except for the absence of the unsaturation. In Table S10, the literature stability constant values determined by various authors for TA binding ability toward Ca^2+^, Mg^2+^, Cu^2+^, Co^2+^, and Zn^2+^ are reported. Markich *et al*.^55^ calculated the stability constant values at infinite dilutions, Campi *et al*.^56^ obtained data in NaClO_4(aq)_ at *I* = 0.10 mol dm^–3^ and *T* = 293.15 K for the five M^2+^, whereas Kiss *et al*.^57^ determined values for the formation constants in NaCl_(aq)_ at *I* = 0.20 mol dm^–3^ and *T* = 298.15 K. In the case of the Cu^2+^ interactions, both the latter authors did not consider metal hydrolysis in the calculations.^56,57^ They reported similar speciation models, with minor discrepancies probably due to the difference in the ionic medium and the uncertain presence of the Cu_2_L species, despite achieving a good fit. To the best of the authors’ knowledge, no values were found for TA *vs* Cd^2+^. In Table S10, for comparison, the data at infinite dilution for *trans*-aconitic acid are given in parentheses. From this preliminary analysis, it can be noticed that the stability constants for *t*AA are slightly higher than those for TA, except for those for Cu^2+^.

In Figure 7 and Table S11, literature stability constant data are listed for six mono-, di-, and tricarboxylic acids toward eight metal cations (Ca^2+^, Mg^2+^, Cu^2+^, Zn^2+^, Co^2+^, Cd^2+^, Pb^2+^, Mn^2+^) at *I* = 0.10–0.15 mol dm^–3^ in various Na^+^ and K^+^ ionic media, and compared with the corresponding *trans*-aconitic acid values.^44,49,58^ The ligands considered are acetic (ACA), tricarballylic (TA) (Table S10), succinic (SA), fumaric (FA), maleic (MA), and citric (CA) acids; their structures are reported in Figure S11. Based on the data currently available, no values were found for MA and FA *vs* Co^2+^ and for SA with Mg^2+^.

**Figure 7:**
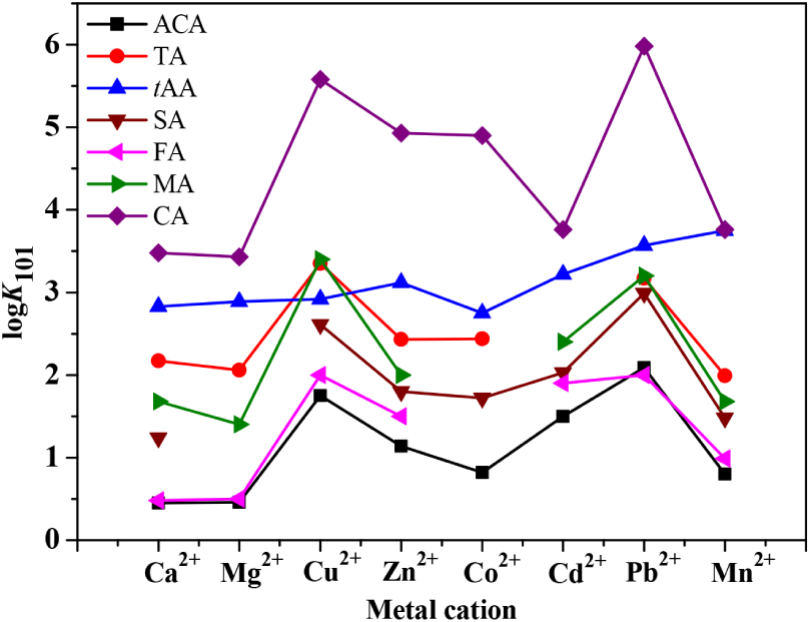
Equilibrium constant trends reported in literature^44,49,58^ for the 1:1 stoichiometry species (ML), where L = acetic (ACA), tricarballylic (TA), *trans*-aconitic (*t*AA), succinic (SA), fumaric (FA), maleic (MA), and citric (CA) acids for various metal cations at *I* = 0.10–0.15 mol dm^–3^ in different Na^+^ and K^+^ ionic media.

As expected, the stability constants of CA are higher than those of *t*AA, possibly owing to the presence of an additional hydroxyl group in the citrate structure and one or two carboxylic groups with respect to the other considered acids (e.g., ACA, SA, FA, MA). By analyzing Figure 5 and Table S11, several other observations can be made.

Some differences between unsaturated and saturated carboxylate ligands were noticed, potentially owing to the higher charge polarization tendency and rigid structure of the former ones. In fact, saturated succinic acid displays intermediate values between fumaric and maleic acids, namely the *trans* and *cis* forms of butenedioic acid, respectively.^59^ Furthermore, MA assumes higher stability constants than FA (in turn, similar data reported with respect to ACA), probably due to the chelate effect, which is not possible for fumarate complexes because of the FA *trans*- configuration. When comparing the formation constant values for all the organic ligands considered, the values with Cu^2+^ were consistently greater than those with the other M^2+^, except for *t*AA, where the log *K*_101_ value for Mn^2+^ is higher than that for other metals. In general, with the exemption of the already discussed citric acid, the stability of *trans*-aconitic acid toward the considered M^2+^ is stronger with respect to the focused literature on mono-, di-, and tricarboxylic acids. This suggests that *t*AA may be more efficient for the remediation of these metals in real matrices to be further investigated.

In the case of Cu^2+^, the pL_0.5_ was calculated for various mono-, di-, saturated and unsaturated tricarboxylic acids (structures in Figure S11) at *I* = 0.10–0.15 mol dm^–3^ in Na^+^ ionic media, pH ∼ 8.1, and *T* = 293.15 and 298.15 K. As observable in Figure 8, the sequestering ability increases by raising the number of carboxylic groups and in the presence of extra *O*-donor sites. Conversely, it decreases as the number of alkyl spacers among the −COOHs increases, while it is slightly favored when no unsaturation is present in the ligand structure. In fact, the Cu^2+^ sequestration is the lowest for acetic acid and the highest for citric acid.

**Figure 8:**
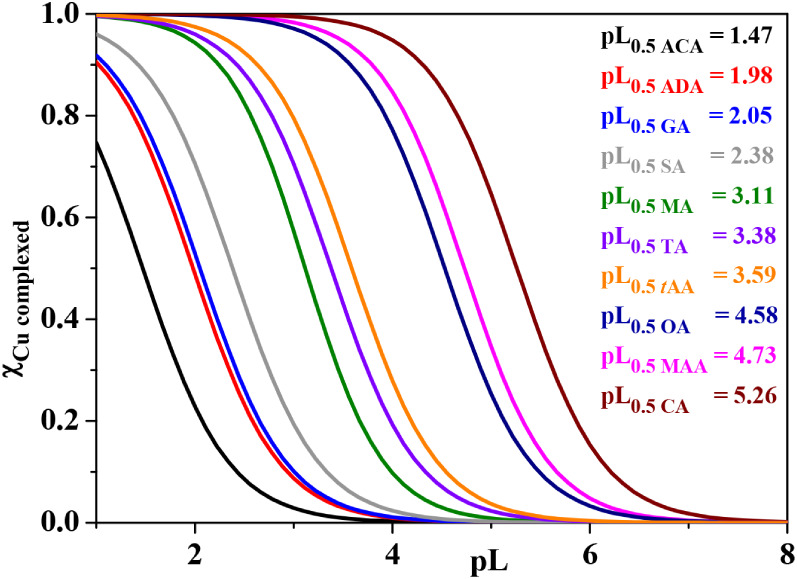
Sequestration diagrams of various mono-, di-, and tricarboxylic acids toward Cu^2+^ at *I* = 0.10–0.20 mol dm^–3^ in Na^+^ ionic media, pH ∼ 8.1, and *T* = 293.15 and 298.15 K.

The similar behavior exhibited by the *trans*-aconitic and the tricarballylic acids toward the sequestration of Cu^2+^ is supported by our DFT-computed binding energies, which are −55.19 kcal mol^–1^ and −56.18 kcal mol^–1^, respectively, for the species ML^–^ shown in Figure 9.

**Figure 9:**
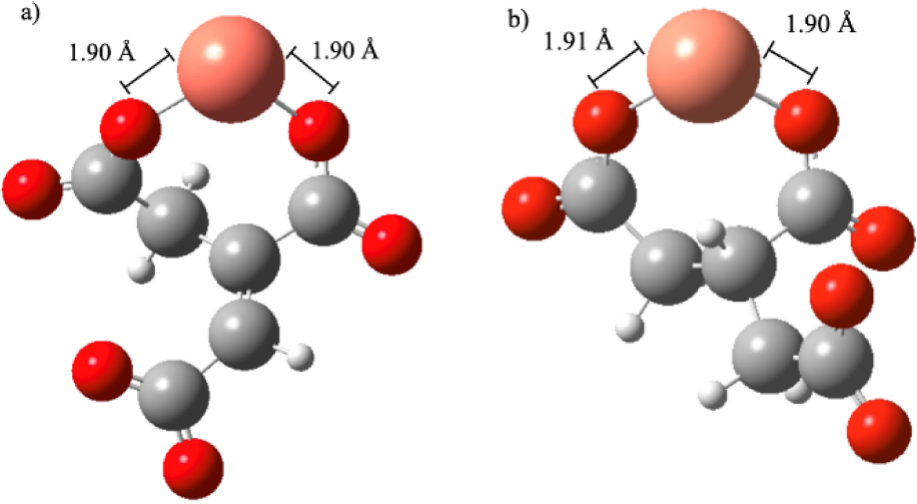
Relaxed molecular geometries of the CuL^–^ complex: a) *trans*-aconitic acid; b) tricarballylic acid.

### 3.5 Speciation Simulation *t*AA in Model of Seawater (SW) and Water for Industrial Use (WIP)

In studies focusing on ligands of biological and environmental relevance, it is frequently crucial to understand their behavior in real systems, such as biological fluids or natural water, multicomponent solutions, usually featured by a low ionic strength (except for seawater).

In this work, two case studies were selected: the first concerns marine water and the second water for industrial purposes.

Once the speciation of *trans*-aconitic in the presence of Ca^2+^, Mg^2+^, Cu^2+^, and Zn^2+^ was known, as well as the corresponding equilibrium constants of the complexes, an evaluation was conducted to examine the ligand distribution in a real system, namely, seawater (SW). The speciation model used as input included all marine water conservative elements, such as Na^+^, K^+^, Ca^2+^, Mg^2+^, Cl^–^, SO_4_^2–^, CO_3_^2–^ while nonconservative ones were neglected. In addition, the interactions of the major seawater components with Cu^2+^ and Zn^2+^, with the *trans*-aconitic acid, as well as the metal cation hydrolysis and *t*AA protonations, were considered. The formation percentages of the species were calculated at typical SW pH (∼8.1).Figure 10a shows the presence of 39% of NaL^2–^ species, as expected, since sodium is one of the major marine water constituents. This is followed by 26% of MgL^–^, and 5% of CaL^–^, owing to the higher Mg^2+^ concentration than Ca^2+^ in the considered natural system, while 30% of the ligand remains in its deprotonated form. These results underline that various species are quite weak, except for the MgL^–^ one; its significant formation percentage, even in the presence of other interfering divalent cations, suggests that it cannot be ignored in the development of removal strategies.

**Figure 10:**
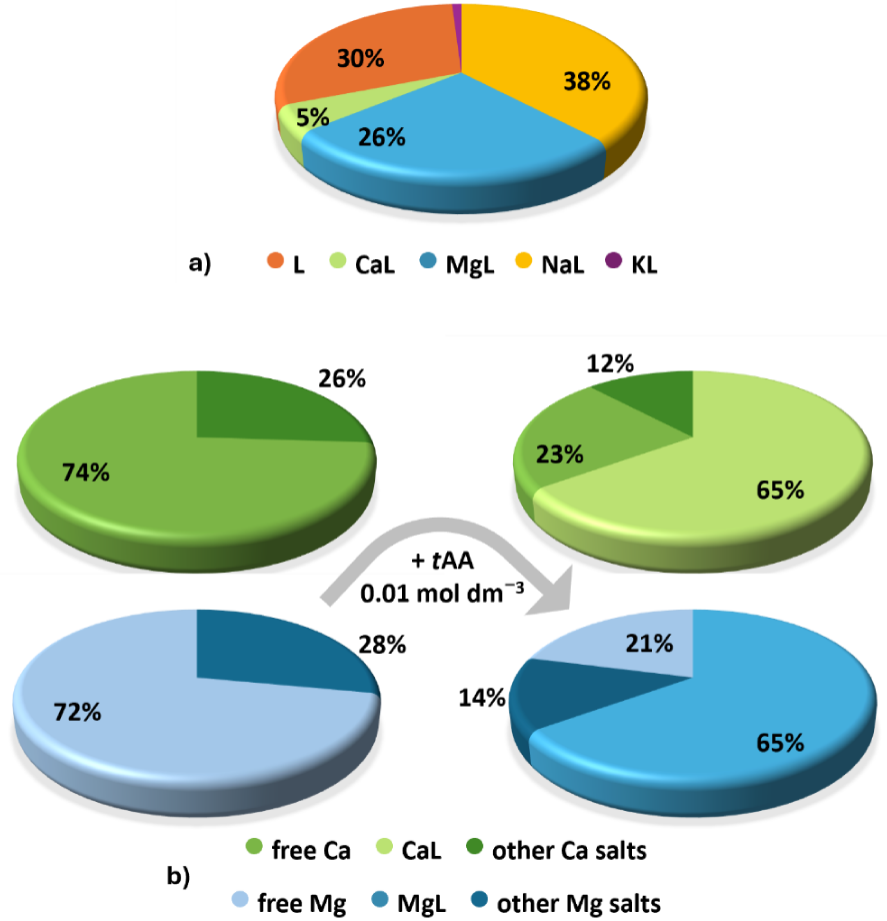
Pie charts of *trans*-aconitic acid (*t*AA) species: a) under seawater conditions (*c*_Na_ = 0.480 mol dm^–3^, *c*_Cl_ = 0.55 mol dm^–3^, *c*_SO4_ = 0.029 mol dm^–3^, *c*_L_ = 0.001 mol dm^–3^, *c*_K_ = 0.01 mol dm^–3^, *c*_Ca_ = 0.01 mol dm^–3^, *c*_Mg_ = 0.043 dm^–3^, *c*_CO3_ = 0.002 mol dm^–3^, *I* ∼ 0.72 mol dm^–3^, *T* = 298.15 K); b) speciation of Ca^2+^ and Mg^2+^ in a WIP at *I* = 0.10 mol dm^–3^, *T* = 298.15 K, in the absence (left) and in the presence (right) of *c* = 0.01 mol dm^–3^ of *t*AA. Charges are omitted for simplicity.

Water for industrial purposes (WIP) must be soft in terms of the Mg^2+^ and Ca^2+^ content. By considering the WIP composition (*c*_Na_ = 0.05 mol dm^–3^, *c*_Ca_ = 0.005 mol dm^–3^, *c*_Mg_ = 0.005 mol dm^–3^, *c*_Cl_ = 0.055 mol dm^–3^, *c*_CO3_ = 0.005 mol dm^–3^, *c*_SO4_ = 0.003 mol dm^–3^, *I* = 0.0885 mol dm^–3^),^60^ the corresponding speciation diagram was built at *I* = 0.10 mol dm^–3^ and *T* = 298.15 K. All interactions among the solution components were considered. At pH ∼ 6.5, Ca^2+^ is generally present as a free cation (74%). Treating the water with *t*AA 0.01 mol dm^–3^, the free metal lowers to 23%, while the 65% of formation of the CaL^–^ species can be observed (Figure 10b). The remaining species are CaSO_4_ and CaCl^+^. In the case of Mg^2+^, the free metal ions decrease from 72% to 21%, forming 65% of MgL^–^ species and decreasing in other Mg salts (Mg­(SO_4_) and MgCl^+^) from 28% to 14%. It is, therefore, evident that *t*AA may be able to interact effectively with Ca^2+^ and Mg^2+^ under these conditions. Based on these considerations, *trans*-aconitic acid could be employed as a potential antiscale agent^61^ and could also serve as a possible greener alternative to typical nonbiodegradable agents such as EDTA,^62^ although it displays lower metal stability and sequestering capability. In this context, the somewhat lower stability of *t*AA complexes compared with that of stronger chelating ligands (e.g., citrate) does not necessarily represent a limitation. Excessively stable complexes may reduce the reversibility of metal binding and complicate subsequent treatment or recovery steps. The moderate complexing ability of *trans*-aconitic acid may therefore provide a suitable compromise between an effective interaction with Ca^2+^ and Mg^2+^ and sufficient flexibility of the treatment process.

## 4 Conclusions

This study explored a potential alternative to conventional, non-biodegradable, or commercial ligands for M^2+^ pollutant remediation and antiscaling applications in natural and industrial waters. In this light, the interaction of *trans*-aconitic acid (*t*AA) with a series of divalent metal cations of environmental and biological relevance, such as Ca^2+^, Mg^2+^, Cu^2+^, Co^2+^, and Zn^2+^, was studied in KCl_(aq)_ at various ionic strength conditions. Potentiometric, UV–vis, and ^1^H NMR were employed for the determination of formation constants. The data analysis confirmed a weak metal–ligand interaction, as expected. Speciation models revealed three complex species (ML^–^, MHL^0^_(aq)_, and MH_2_L^+^) for Ca^2+^, Mg^2+^, and Zn^2+^; two species (CoL^–^ and CoHL^0^_(aq)_) for Co^2+^; and a single complex (CuL^–^) for Cu^2+^. Furthermore, Density Functional Theory (DFT) calculations allowed us to achieve a better understanding of the microscopic acidity of the three sites undergoing deprotonation reactions, indicating the most/least likely sites for deprotonation, though some discrepancies were noticed with respect to NMR experiments, likely due to the implicit solvent approximation here adopted. Then, another series of DFT calculations was also performed to investigate the interaction between the divalent cations, such as Ca^2+^ and Mg^2+^, with the *trans*-aconitate ligand to form the MH_2_L^+^ and ML^–^ complexes. It turns out that although Ca^2+^ and Mg^2+^ behave similarly to each other, different chelation sites are afforded because of a sizably different ionic radius, providing a plausible explanation for the experimentally observed discrepancies. DFT-based calculations of the binding energies between L^3–^ (the most abundant form of *trans*-aconitic acid at pH ∼ 8.1) and Ca^2+^, Mg^2+^, and Cu^2+^ to form the ML^–^ species gain additional light on the reason why L^3–^ better sequestrates Cu^2+^ and Mg^2+^ metal cations with respect to Ca^2+^. All of these findings combined with the dependence of the equilibrium constants on ionic strength provide a comprehensive thermodynamic picture linked to molecular-level observations.

The sequestering ability and affinity of *t*AA toward M^2+^ cations, as well as simulations in real conditions, were evaluated and carried out as a function of the pL_0.5_ and pM parameters, species formation percentages, at different pH and ionic strengths, with particular attention to those that simulate seawater and waters for industrial-purpose conditions. Although citric acid generally forms more stable complexes with divalent metal cations than *trans*-aconitic acid, the present results show that *t*AA is still able to effectively interact with relevant metal ions under environmentally and industrially relevant conditions. In this perspective, the moderate complex stability of *t*AA acid may represent a suitable compromise between efficient metal binding and sufficient reversibility of the interaction, which can be advantageous in applications such as water treatment and antiscaling processes.

Based on the obtained results, although literature data comparison showed that citric acid generally proves to be more effective than *trans*-aconitic acid, this study nonetheless provides valuable insights into the *t*AA speciation and affinity for divalent cations. In particular, the data indicate that, with the CA exception, the investigated ligand demonstrates greater stability toward M^2+^ cations compared to several mono-, di-, and tricarboxylic acids reported in the literature. In addition, *t*AA forms more stable metal complexes than its analogue, tricarballylic acid, suggesting a potentially more effective use for the remediation of these metals in real matrices. These results contribute to a broader evaluation of sustainable alternatives for water treatment, particularly under specific environmental and industrial conditions. Further research could explore the applicability of *t*AA in targeted scenarios where its properties may offer advantages, as well as investigate its behavior in complex matrices to better understand its effectiveness compared to conventional chelating agents.

## Supplementary Material



## Notes

Details on the experimental procedures, NMR signal assignment, and determination of p*K*_a_ values from chemical shift titration and dδ/dpH analysis; tables and figures related to thermodynamic, data UV–vis, and ^1^H NMR spectra at different experimental conditions (PDF)

## Notes

Dedicated to my beloved grandmother, Caterina Soccorsa Adamo, and my dear nephew, Lorenzo.
