# Supplementary material for: Exploring the Speciation of trans-Aconitic Acid in Aqueous Solution: Interactions with Divalent Metal Cations of Environmental Relevance
Source: ACS Omega. 2026 May 4;11(19):28737–49. doi: 10.1021/acsomega.6c01283 (PMC13191524; doi:10.1021/acsomega.6c01283)
Supplement: Supplementary file 1 [file ao6c01283_si_001.pdf]

Supplementary Information

Exploring the speciation of *trans*-aconitic acid in aqueous solution. Interactions with divalent metal cations of environmental relevance.

Anna Irto[a#], Rosita Cappai[b#], Alessandro Amadeo[a,c], Massimiliano Peana[b], Giuseppe Cassone[d], Concetta De Stefano, Clemente Bretti[a]*.

[a] Department of Chemical, Biological, Pharmaceutical and Environmental Sciences, University of Messina, Viale Ferdinando Stagno d'Alcontres, 31, I-98166 Messina, Italy.

[b] Department of Chemical, Physical, Mathematical and Natural Sciences (DISCI), University of Sassari, via Vienna 2, 07100, Sassari, Italy.

[c] Department of Chemistry, Biology and Biotechnologies, University of Perugia, Via dell'Elce di sotto, 8, 06123, Perugia, Italy.

[d] Institute for Chemical-Physical Processes, National Research Council of Italy (CNR-IPCF), Viale Ferdinando Stagno d'Alcontres 37, 98158 Messina (Italy).

*Correspondence to: cbretti@unime.it

# These authors contributed equally to the manuscript

List of Symbols

e.m.f. Electromotive force

E^0 Standard electrode potential

j_a Liquid junction potential coefficient

pK_W Ionic product of water

$\log K_i^H$ i^{th} Protonation constant

I Ionic strength (in mol dm^{-3})

T temperature in Kelvin

C_{ik} Ionic strength dependence parameter expressed in the molar scale

U Error function

$pL_{0.5}$ Metals sequestering parameter

pM Metals affinity parameter

$p^* = \sum p^*_{reactants} - \sum p^*_{products}$ (p^* = stoichiometric coefficient of the components)

*t*AA, *trans*-aconitic acid

TA Tricarballylic Acid

CA Citric Acid

SA Succinic Acid

FA Fumaric Acid

MA Maleic Acid

ACA ACetic Acid

ADA ADipic Aacid

GA Glutaric Acid

OA Oxalic Acid

MAA Malonic Acid

UV-Vis UltraViolet-Visible

^1H NMR proton nuclear magnetic resonance

^{13}C NMR carbon nuclear magnetic resonance

DFT Density Functional Theory

D.H. Debye-Hückel

$M_j(OH)_i^{(2j-i)}$ metals hydrolytic species

Details on the experimental procedures

Chemicals

Fresh potassium hydroxide (KOH) and hydrochloric acid (HCl) solutions were prepared from concentrated stocks and standardized against potassium hydrogen phthalate (for bases) and sodium carbonate (for acids), both pre-dried in an oven at T = 383.15 K for 2 hours. Strong base solutions were stored in dark bottles with soda lime traps. The concentration of *trans*-aconitic acid in the solutions was determined using alkalimetric titrations. Potassium chloride (KCl) solutions were prepared by weighing the solid, which had been previously dried in an oven at T = 383.15 K for 2 hours. Metal chloride salts in various hydrated forms were weighed for the solutions preparation and standardized against EDTA. Glassware and twice-distilled water (resistivity ≥ 18 MΩ cm) were used for the preparation of the solutions. Further details are listed in Table S1.

Solution studies

Potentiometric titrations were carried out utilizing a Metrohm (Herisau, Switzerland) 809 Titrando apparatus, which consists of an automatic burette (total volume 10 cm^3) and a combined glass electrode (Metrohm, model 6.0262.100). The standard uncertainty was u(E) = ± 0.2 mV for electromotive force (*e.m.f.*) measurements and u(V) = ± 0.003 cm^3 for titrant volume readings. A PC was interfaced with the apparatus, and the experiments control was managed through a Tiamo 2.5 software, facilitating the precise regulation of titrant delivery, data acquisition, and the monitoring of *e.m.f.* stability. The potentiometric measurements were carried out at T = 298.15 K, at different ionic strengths ($0.1 \leq I$/mol $dm^{-3} \leq 1.0$), metal and ligand concentrations, and molar ratios. The potentiometric titrations were conducted with continuous magnetic stirring, and the solutions exposed to nitrogen gas ($N_{2(g)}$), previously saturated with water to eliminate and prevent the dissolution of oxygen ($O_{2(g)}$) and carbon dioxide gases ($CO_{2(g)}$). To determine the stability constants of the M^{2+}/tAA systems, 25 cm^3 of titrant solution containing suitable amounts of ligand (c_L), $HCl_{(aq)}$ (c_H) to regulate the starting pH at ~ 2.0, $KCl_{(aq)}$ to reach the desired I/mol dm^{-3} value, and metal cation (c_M), were added into the measurement cell. The solutions were titrated with CO_2 free standard base ($KOH_{(aq)}$) solutions up to pH ~ 10.0, or until the electrode started drifting towards lower pH values which indicated the onset of precipitation of sparingly soluble species, hardly detectable by the naked eye in the initial state. Before each measurement, the electrode was calibrated in terms of free hydrogen ion concentration [H^+] (not activity) by means of a strong acid (HCl) - strong base

($KOH_{(aq)}$) titrations in the same pH and ionic strength conditions of the experiment. The parameters obtained from these titrations, namely E^0 and $\log K_W$ were used to convert the measured potential (*e.m.f.*) into pH on a free scale, employing the formula pH = − log[H^+]. A thermocryostat, model D1-G Haake (uncertainty ± 0.1 K) was used to perform measurements at constant temperature of 298.15 K. The reproducibility of the experiments was ensured by two different operators using various electrodes (glass and reference ones), reagent solutions and moreover potentiometric systems.

Spectrophotometric titrations were carried out by means of a Varian (Agilent Scientific instruments, California, U.S.A.) Cary 50 UV-Vis spectrophotometer, equipped with an optic fiber probe (path length 1 cm). All the apparatus were controlled by the Varian Cary WinUV software. The titrant solutions were delivered by means of an automatic burette (total volume 10 cm^3), Metrohm (Herisau, Switzerland) 665 model. The same solutions contained a combined Ross type 8102 glass electrode (Thermo-Orion, Waltham, MA U.S.A), connected to a Metrohm potentiometer, allowing the contemporary measurements of the *e.m.f.* The ligand protonation constants and the molar extinction coefficients (ε/mol^{-1} dm^3 cm^{-1}) values for the protonated and free species were determined at T = 298.15 K and in $KCl_{(aq)}$ at I = 0.15 mol dm^{-3} in the pH range 2.0-10.5 and 200 ≤ λ/nm ≤ 800. The binding ability of *trans*-aconitic acid towards the different metal cations was studied at the same experimental conditions.

^{1}H NMR and ^{13}C NMR spectra of *t*AA and M^{2+}/*t*AA complexes (M^{2+} = Ca^{2+}, Mg^{2+} and Zn^{2+}) at I = 0.15 mol dm^{-3} in $KCl_{(aq}$ and and T = 298.15 K , were recorded in 9:1 (v/v) H_2O:D_2O mixture on a Bruker (Billerica, Massachusetts, U.S.A.) Ascend 400 MHz spectrometer operating with a 5 mm automated tuning and matching broad band fluorine observation probe (BBFO) with z-gradients. For the ligand titration, the stock *t*AA solution was prepared at c_{tAA} = 5 mmol dm^{-3} and 100 mmol dm^{-3} concentration, respectively for ^{1}H and ^{13}C, and acidified with 2 equivalents of hydrochloric acid. For the metal complex titrations, the stock M^{2+}/*t*AA solution was prepared at 1:1 metal:ligand molar ratio at c_{tAA} = 2 mmol dm^{-3} and 100 mmol dm^{-3} concentration, respectively for ^{1}H and ^{13}C, and 1:3 molar ratio at c_{tAA} = 5 mmol dm^{-3}, both acidified with 2 equivalents of hydrochloric acid. The pH was set according to those of maximum formation of the selected species in distribution curves and measured by Orion (Espoo, Finland) 520 pH meter equipped with CRISON (Barcelona, Spain) 2000 combined micro electrode. Further details on the component's concentration employed for the potentiometric, UV-Vis and ^{1}H NMR and ^{13}C NMR measurements are reported in Table S2.

Softwares

The ESAB2M software[1], employing a non-linear least squares method, was utilized to determine parameters from acid-base titrations, such as E^0, $\log K_w$ and j_a, along with the analytical concentrations of the reagents. Experimental potentiometric data, titrant volume (cm^3) *vs e.m.f.* (mV), together with the electrode calibration parameters (E^0 and $\log K_W$), were processed together with the mass balance equation of each component involved into the equilibria by using the BSTAC software[2], while UV-Vis spectrophotometric data were processed with HypSpec 2008[3]. For all collected ^{1}H NMR and ^{13}C NMR solution spectra, where proton exchange reactions were confirmed to be fast on the NMR time scale, the HypNMR program[3] was employed to refine individual chemical shifts of each equilibrium species and calculate the corresponding stability constants by analyzing the average observed chemical shifts. The non-linear least square computer program LIANA[4] was used to fit different equations. Finally, HySS software[5] was used to calculate metal-ligand complex formation percentages and generate distribution diagrams. Throughout the paper, uncertainties are given as a 95 % confidence interval.

NMR Signal Assignment and Determination of pKa Values from Chemical Shift Titration and dδ/dpH Analysis

The assignment of all ^{1}H NMR and ^{13}C NMR resonances of *trans*-aconitic acid was achieved using a combination of one-dimensional ^{1}H and ^{13}C NMR experiments and two-dimensional HSQC and HMBC spectra, allowing unambiguous correlation of proton and carbon sites across the molecular framework.

Numerical derivatives of the chemical shifts with respect to pH (dδ/dpH) were calculated using a central finite-difference scheme implemented via the gradient operator, providing a modelindependent visualization of pH regions associated with enhanced chemical shift sensitivity and proton dissociation events. The pH dependence of each chemical shift was modelled using a triprotic Henderson–Hasselbalch equation:

$$\delta(\mathrm{pH}) = \delta_0 + \frac{\delta_1 - \delta_0}{1 + 10^{\mathrm{p}K_{a1} - \mathrm{pH}}} + \frac{\delta_2 - \delta_1}{1 + 10^{\mathrm{p}K_{a2} - \mathrm{pH}}} + \frac{\delta_3 - \delta_2}{1 + 10^{\mathrm{p}K_{a3} - \mathrm{pH}}}$$

in which the observed chemical shift is expressed as a population-weighted average of the four successive protonation states. Nonlinear least-squares fitting was performed using the Levenberg-Marquardt algorithm, and standard deviations of the fitted $\mathrm{p}K_a$ values were obtained from the diagonal elements of the covariance matrix. Analytical expressions for dδ/dpH were

obtained by differentiating the Henderson–Hasselbalch equation with respect to pH, yielding the sum of three bell-shaped functions centred at the corresponding pK_a values, which were evaluated using the optimized fit parameters.

Tables

Table S1. Chemicals used in this work, purchased from Merck (Darmstadt, Germany). Purity (mass) as stated by the supplier.

Chemical	Formula	CAS n°	Assay (mass)
Potassium chloride	KCl	7447-40-7	≥ 99%
Hydrochloric acid	HCl	7647-01-0	≥ 99%
Potassium hydroxide	$NaOH$	1310-73-2	≥ 99%
Potassium phthalate monobasic	$C_8H_5O_4K$	877-24-7	≥ 99.95%
Sodium carbonate	Na_2CO_3	497-19-8	99.995%
Zinc Chloride	$ZnCl_2$	7646-85-7	> 98%
Magnesium chloride hexahydrate	$MgCl_2 \cdot 6H_2O$	7791-18-6	≥ 99%
Cobalt(II) chloride hexahydrate	$CoCl_2 \cdot 6H_2O$	7791-13-1	≥ 99%
Cupric chloride dihydrate	$CuCl_2 \cdot 2H_2O$	10125-13-0	≥ 99%
Calcium chloride dihydrate	$CaCl_2 \cdot 2H_2O$	10035-04-8	≥ 99%
Deuterium oxide	D_2O	7789-20-0	≥ 99%
Trans-aconitic acid	$C_6H_6O_6$	4023-65-8	> 98%

Table S2. Summary of the components concentrations employed for potentiometric, UV-Vis and ^{1}H and ^{13}C NMR titrations performed in $KCl_{(aq)}$ at T = 298.15 K.

System	Analytical technique	c_{HCl} a)	c_M a)	c_L a)
Ca^{2+}/L^{3-}	Potentiometry	10.0	1.0-3.0	2.0-7.0
	UV-Vis spectrophotometry	10.0	0.02-0.06	0.02-0.06
	^{1}H NMR spectroscopy	10.0	1.6-2.0	2.0-5.0
Mg^{2+}/L^{3-}	Potentiometry	10.0	1.0-2.0	1.0-6.0
	UV-Vis spectrophotometry	10.0	0.02-0.06	0.02-0.06
	^{1}H NMR spectroscopy	10.0	1.6-2.0	2.0-5.0
	^{13}C NMR spectroscopy	-	100	100
Zn^{2+}/L^{3-}	Potentiometry	10.0	1.0-3.0	1.0-4.0
	UV-Vis spectrophotometry	10.0	0.01-0.05	0.02-0.06
	^{1}H NMR spectroscopy	10.0	1.6-2.0	2.0-5.0
Cu^{2+}/L^{3-}	Potentiometry	10.0	1.0-3.0	2.0-5.0
	UV-Vis spectrophotometry	10.0	0.02-0.06	0.02-0.06
Co^{2+}/L^{3-}	Potentiometry	10.0	1.0-3.0	1.0-4.0
	UV-Vis spectrophotometry	10.0	0.01-0.05	0.02-0.06

a) analytical concentration (mmol dm^{-3}); standard uncertainties (u): $u(T)$ = 0.1 K; $u(I)$ = 0.001 mol dm^{-3}; $u(c_H) = u(c_L) = u(c_M) = 10^{-2}$ mmol dm^{-3}.

Table S3. Calculated chemical shifts for the protonated and complex species obtained by ^{1}H NMR titrations at $I = 0.15$ mol dm^{-3} in $KCl_{(aq)}$ and $T = 298.15$ K.

Species	$\delta_{CH\ 1}$	$\delta_{CH_2\ 3}$
L^{3-}	6.50 ± 0.06 [a)]	3.36 ± 0.04 [a)]
HL^{2-}	6.66 ± 0.06	3.48 ± 0.04
H_2L^{-}	6.84 ± 0.06	3.62 ± 0.04
$H_3L^{0}{}_{(aq)}$	6.96 ± 0.06	3.80 ± 0.04
CaL^{-}	6.49±0.03 [a)]	3.36±0.03 [a)]
$CaHL^{0}{}_{(aq)}$	6.77±0.03	3.47±0.03
CaH_2L^{+}	7.06±0.03	3.92±0.03
MgL^{-}	6.48±0.09 [a)]	3.34±0.01 [a)]
$MgHL^{0}{}_{(aq)}$	6.61±0.08	3.43±0.01
MgH_2L^{+}	6.88±0.08	3.70±0.01
ZnL^{-}	6.41±0.03 [a)]	3.36±0.02 [a)]
$ZnHL^{0}{}_{(aq)}$	6.73±0.03	3.46±0.02
ZnH_2L^{+}	6.92±0.03	3.75±0.02

[a)] ±s (standard deviation).

Table S4. pH-dependent variation of the experimental ^{13}C chemical shift values for A, B, C, , 1, 2 and 3 nuclei for *trans*-aconitic acid protonation.

Carbon/pH	1.91	2.00	2.16	2.25	2.42	2.57	2.72	2.82	2.94	3.03	3.15	3.26	3.43	3.60	3.70
A	174.835	174.839	174.923	174.929	175.003	175.092	175.169	175.243	175.344	175.344	175.426	175.554	175.709	175.855	175.921
B	169.608	169.618	169.847	169.873	170.095	170.334	170.548	170.762	171.003	171.027	171.241	171.545	171.896	172.203	172.340
C	168.876	168.888	169.183	169.220	169.512	169.816	170.095	170.365	170.657	170.697	170.963	171.320	171.727	172.072	172.220
2	139.025	139.011	138.828	138.786	138.588	138.399	138.211	138.042	137.896	137.844	137.692	137.524	137.363	137.245	137.210
1	130.619	130.626	130.814	130.832	131.007	131.186	131.342	131.492	131.635	131.661	131.784	131.926	132.063	132.147	132.177
3	33.371	33.371	33.510	33.514	33.634	33.766	33.882	33.999	34.144	34.143	34.264	34.442	34.655	34.836	34.924

Carbon/pH	3.85	3.96	4.15	4.26	4.36	4.54	4.75	4.99	5.31	5.72	5.96	6.23	6.80	7.06	7.32
A	176.107	176.278	176.401	176.540	176.709	176.922	177.152	177.518	178.059	178.654	179.024	179.371	179.640	179.706	179.713
B	172.702	173.007	173.238	173.473	173.706	173.992	174.262	174.584	174.994	175.395	175.636	175.86	176.03	176.072	176.074
C	172.611	172.931	173.178	173.419	173.652	173.939	174.208	174.516	174.899	175.267	175.487	175.687	175.841	175.877	175.881
2	137.120	137.078	137.034	137.018	137.049	137.079	137.145	137.333	137.666	138.062	138.317	138.553	138.740	138.782	138.791
1	132.232	132.259	132.271	132.270	132.259	132.222	132.152	132.022	131.785	131.499	131.319	131.169	131.041	131.013	130.997
3	35.150	35.346	35.490	35.640	35.803	35.992	36.171	36.390	36.678	36.961	37.134	37.296	37.419	37.449	37.452

Table S5. Hydrolysis constants[a)] of the divalent metal cations at various ionic strengths and T = 298.15 K.

I/mol dm^{-3}	logβ_{MOH}	logβ_{MOH}
	Ca^{2+}	Mg^{2+}
0.15	−12.91	−11.61
0.50	−12.96	−11.64
0.75	−12.97	−11.66
1.00	−12.98	−11.67

I/mol dm^{-3}	logβ_{MOH}	log$\beta_{M(OH)_2}$	log$\beta_{M(OH)_3}$	log$\beta_{M(OH)_4}$	log$\beta_{M_2(OH)_2}$	
			Cu^{2+}			
0.15	−8.242	−17.555	−27.806	−39.053	−10.633	
0.50	−8.298	−17.623	−27.820	−38.833	−10.743	
0.75	−8.287	−17.624	−27.830	−38.771	−10.774	
1.00	−8.261	−17.611	−27.840	−38.738	−10.791	
			Co^{2+}			
0.15	−9.883	−19.090	−31.53			
0.50	−9.953	−19.152	−31.60			
0.75	−9.967	−19.189	−31.64			
1.00	−9.970	−19.217	−31.69			
			Zn^{2+}			log$\beta_{M_2(OH)_6}$
0.15	−9.206	−17.138	−28.378	−40.673	−8.749[b)]	−57.511
0.50	−9.261	−17.176	−28.325	−40.459	−8.682	−57.357
0.75	−9.263	−17.166	−28.288	−40.380	−8.671	−57.284
1.00	−9.255	−17.146	−28.251	−40.321	−8.670	−57.221

[a)] equilibrium: $j\ M^{2+} + i\ H_2O = M_j(OH)_i^{(2j-i)} + i\ H^+$; [b)] species $Zn_2(OH)$.

Table S6. Overall[a)] and stepwise[b)] formation constants determined by potentiometry at $I = 0.15$ mol dm^{-3} in $KCl_{(aq)}$, $T = 298.15$ K and details on the maximum formation percentages and pHs of the various M^{2+}/L^{3-} species (analytical concentrations: c_M = 1 mmol dm^{-3}; c_L = 3 mmol dm^{-3}).

Species	$\log\beta_{ijk}$	$\log K_{ijk}$	Max(%)	pH_{max}
CaL^-	2.83 ± 0.01 [c)]	2.83	60	8.0
$CaHL^0_{(aq)}$	7.79 ± 0.02	2.20	21	4.5
CaH_2L^+	11.44 ± 0.05	1.88	13	3.0
MgL^-	2.89 ± 0.02 [c)]	2.89	60	8.0
$MgHL^0_{(aq)}$	8.08 ± 0.03	2.47	30	4.7
MgH_2L^+	11.94 ± 0.04	2.38	28	3.0
ZnL^-	3.12 ± 0.03 [c)]	3.12	70	6.0
$ZnHL^0_{(aq)}$	8.33 ± 0.04	2.72	40	4.0
ZnH_2L^+	12.06 ± 0.06	2.50	30	3.0
CuL^-	2.92 ± 0.01 [c)]	2.92	60	6.0
CoL^-	2.75 ± 0.04 [c)]	2.75	45	6.0
$CoHL^0_{(aq)}$	8.01± 0.06	2.40	30	4.6

[a)] $\log\beta_{ijk}$ related to equilibrium in eq. 2; [b)] $\log K_{ijk}$ related to equilibrium in eq. 3; [c)] ±s (standard deviation); standard uncertainties: $u(T) = 0.1$ K, $u(\text{Max}) = 1\%$; $u(\text{pH}) = 0.01$.

Table S7. Overall[a),b)] formation constants determined by potentiometry for the various M^{2+}/L^{3-} species (charges omitted for simplicity) at different ionic strengths and $T = 298.15$ K.

species	CaL	CaHL	CaH_2L
I/mol dm^{-3}	logK_{110}	logβ_{111}	logβ_{112}
0.25	2.55	7.46	11.13
0.50	2.06	6.91	10.60
0.75	1.66	6.46	10.17
1.00	1.26	6.01	9.74

species	MgL	MgHL	MgH_2L
I/mol dm^{-3}	logK_{110}	logβ_{111}	logβ_{112}
0.25	2.62	7.77	11.64
0.50	2.16	7.24	11.11
0.75	1.78	6.81	10.69
1.00	1.41	6.39	10.28

species	ZnL	ZnHL	ZnH_2L
I/mol dm^{-3}	logK_{110}	logβ_{111}	logβ_{112}
0.25	2.97	8.16	11.84
0.50	2.85	8.04	11.60
0.75	2.88	8.08	11.54
1.00	2.97	8.20	11.54

species	CuL
I/mol dm^{-3}	logK_{110}
0.25	2.70
0.50	2.44
0.75	2.32
1.00	2.26

species	CoL	CoHL
I/mol dm^{-3}	logK_{110}	logβ_{111}
0.25	2.61	7.81
0.50	2.54	7.64
0.75	2.62	7.64
1.00	2.77	7.71

[a)] logβ_{ijk} related to equilibrium in eq. 2; [b)] s (standard deviation) ± 0.02-0.04.

Table S8. Overall[a)] and stepwise[b)] formation constants determined by ^{1}H and ^{13}C NMR spectroscopy at $I = 0.15$ mol dm^{-3} in $KCl_{(aq)}$, $T = 298.15$ K.

	^{1}H NMR		^{13}C NMR	
Species	$\log\beta_{ijk}$	$\log K_{ijk}$	$\log\beta_{ijk}$	$\log K_{ijk}$
CaL^{-}	2.88 ± 0.07 [c)]	2.88	-	-
$CaHL^{0}_{(aq)}$	8.08 ± 0.02	2.44	-	-
CaH_2L^{+}	11.68 ± 0.02	2.06	-	-
MgL^{-}	2.79 ± 0.12 [c)]	2.79	3.26 ± 0.02 [c)]	3.26
$MgHL^{0}_{(aq)}$	8.29 ± 0.15	2.65	8.13 ± 0.03	2.64
MgH_2L^{+}	11.86 ± 0.30	2.24	11.25± 0.03	1.80
ZnL^{-}	2.95 ± 0.14 [c)]	2.95	-	-
$ZnHL^{0}_{(aq)}$	8.40 ± 0.16	2.76	-	-
ZnH_2L^{+}	12.08 ± 0.16	2.46	-	-

[a)] $\log\beta_{ijk}$ related to equilibrium in eq. 2; [b)] $\log K_{ijk}$ related to equilibrium in eq. 3; [c)] ±s (standard deviation); standard uncertainties: $u(T) = 0.1$ K.

Table S9. $pL_{0.5}$ values determined using eq. 5 for the different M^{2+}/L^{3-} systems at various experimental conditions in $KCl_{(aq)}$ and $T = 298.15$ K.

M^{2+}	I/mol dm^{-3}	pH	$pL_{0.5}$	M^{2+}	I/mol dm^{-3}	pH	$pL_{0.5}$
Ca^{2+}	0.15	2.0	1.02	Ca^{2+}	0.72	8.1	2.65
	0.15	3.0	1.69		1.00	8.1	2.70
	0.15	4.0	2.06	Mg^{2+}	0.15	7.4	2.56
	0.15	5.0	2.36		0.72	8.1	2.96
	0.15	6.0	2.67	Zn^{2+}	0.15	7.4	3.11
	0.15	7.4	2.76		0.72	8.1	2.83
	0.15	8.1	2.77	Cu^{2+}	0.15	7.4	3.25
	0.15	9.0	2.77		0.72	8.1	3.71
	0.15	10.0	2.77	Co^{2+}	0.15	2.0	< 0.10
	0.25	8.1	2.67		0.15	3.0	0.82
	0.50	8.1	2.62		0.15	4.0	1.71
	0.72	2.0	1.22		0.15	6.0	2.22
	0.72	3.0	1.90		0.15	7.4	2.29
	0.72	4.0	2.27		0.15	8.1	2.28
	0.72	5.0	2.47		0.72	8.1	2.35
	0.72	6.0	2.62				
	0.72	7.4	2.65				

Table S10. Overall stability constants reported in the literature for various M^{2+}/TA systems.

I/mol dm^{-3}	Metal cation	ML[a]	MHL	MH_2L	M_2L	MLOH
→ 0[a)]	Ca^{2+}	3.33 (4.34)	8.74 (9.55)	12.57 (13.18)	4.46	
0.10[b)]	Ca^{2+}	2.17	6.77	10.53		
→ 0[a)]	Mg^{2+}	3.34 (4.36)	8.50 (9.79)	12.48 (13.64)	4.21	
0.10[b)]	Mg^{2+}	2.06	6.64	10.59		
→ 0[a)]	Cu^{2+}	4.72 (4.34)	9.91	13.19	6.99	−1.77
0.10[b)]	Cu^{2+}	3.70	8.46	11.83	5.30	−4.34
0.20[c)]	Cu^{2+}	3.35	8.03	11.53	4.87	−3.34
→ 0[a)]	Zn^{2+}	3.70 (4.45)	8.95 (9.88)	12.73(13.67)		
0.10[b)]	Zn^{2+}	2.43	6.81	10.46		
0.10[b)]	Co^{2+}	2.44 (4.07)	6.84 (9.61)	10.51		

[a)] at infinite dilution,[6] $T = 298.15$ K; [b)] in $NaClO_{4(aq)}$,[7] $T = 293.15$ K; [c)] in $KCl_{(aq)}$,[8] $T = 298.15$; in parentheses the corresponding data determined for tAA interaction with M^{2+}.

Table S11. Stability constants reported in the literature[9-11] for the ML species for various M^{2+}/mono-, di- and tricarboxylic acids systems at I = 0.10-0.15 mol dm^{-3} in various Na^{+} and K^{+} ionic media and T = 298.15 K.

Metal cation	Acetic Acid (ACA)	Tricarballylic Acid (TA)	*Trans*-aconitic acid (*t*AA)	Succinic acid (SA)	Fumaric acid (FA)	Maleic acid (MA)	Citric Acid (CA)
Ca^{2+}	0.45 [a)]	2.17 [b)]	2.83 [c)]	1.24 [d)]	0.48 [c)]	1.68 [c), g)]	3.48 [d)]
Mg^{2+}	0.46 [a)]	2.06 [b)]	2.89 [c)]	-	0.50 [c), g)]	1.40 [c), g)]	3.43 [d)]
Cu^{2+}	1.75 [e)]	3.35 [b)]	2.92 [c)]	2.61 [f)]	2.00 [c), g)]	3.40 [c)]	5.58 [a)]
Zn^{2+}	1.14 [e)]	2.43 [b)]	3.12 [c)]	1.80 [f)]	1.50 [c), g)]	2.00 [c)]	4.93 [d)]
Co^{2+}	0.82 [e)]	2.44 [b)]	2.75 [c)]	1.72 [d)]	-	-	4.90 [d)]
Cd^{2+}	1.50 [h)]	-	3.22 [c)]	2.03 [d)]	1.90 [c), g)]	2.40 [c), g)]	3.76 [d)]
Pb^{2+}	2.09 [h)]	3.17 [b)]	3.57 [c)]	2.99	2.00 [c)]	3.20 [c)]	5.98 [f)]
Mn^{2+}	0.80 [e)]	1.99 [b)]	3.75 [c)]	1.48 [d)]	0.99 [c)]	1.68 [c)]	3.76 [d)]

[a)] I = 0.15 mol dm^{-3} in $NaCl_{(aq)}$; [b)] I = 0.10 mol dm^{-3} in $NaClO_{4(aq)}$ and T = 293.15 K; [c)] I = 0.10 mol dm^{-3} in $KCl_{(aq)}$; [d)] I = 0.10 mol dm^{-3} in Na^{+} ionic medium; [e)] I = 0.10 mol dm^{-3} in $NaCl_{(aq)}$; [f)] I = 0.10 mol dm^{-3} in $NaClO_{4(aq)}$; [g)] estimated value; [h)] I = 0.10 mol dm^{-3} in $KNO_{3(aq)}$.

Figures

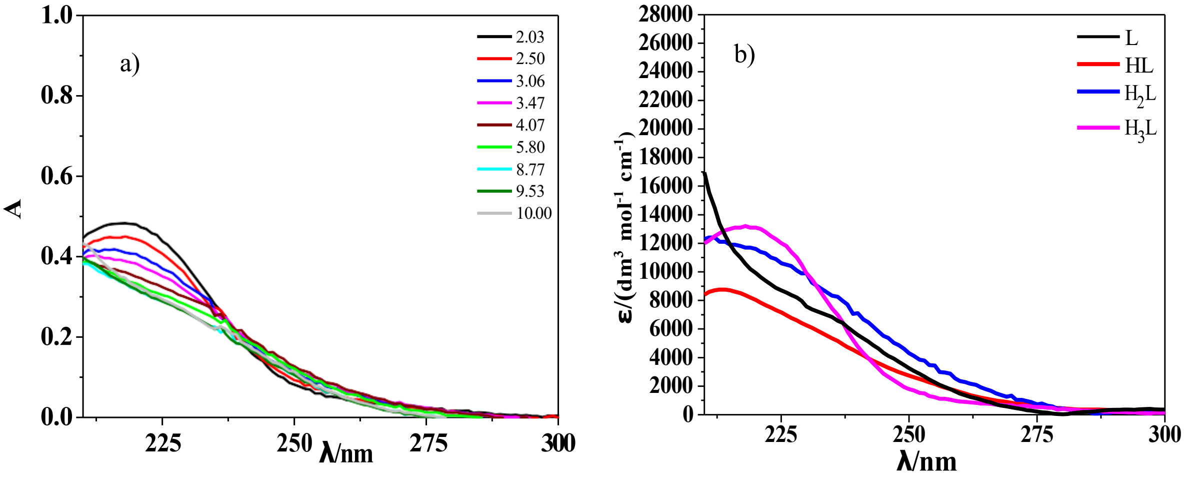

Figure S1. Spectrophotometric scans of a) *trans*-aconitic acid *vs* pH at c_L = 0.04 mmol dm^{-3} and b) graphical representation of molar absorptivities of the ligand species at I = 0.15 mol dm^{-3} in $KCl_{(aq)}$ and T = 298.15 K.

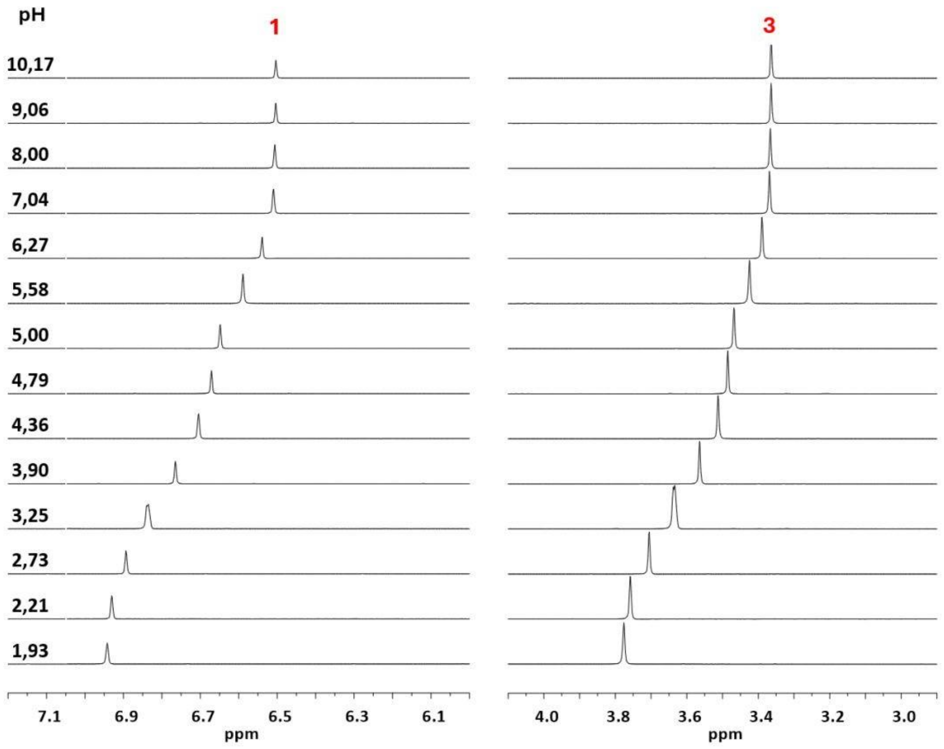

Figure S2 ^{1}H NMR spectra of *trans*-aconitic acid recorded in 9:1 (v/v) H_2O/D_2O mixture over the pH range 1.93-10.17 at $c_L = 5$ mmol dm^{-3}, $I = 0.15$ mol dm^{-3} in $KCl_{(aq)}$ and $T = 298.15$ K.

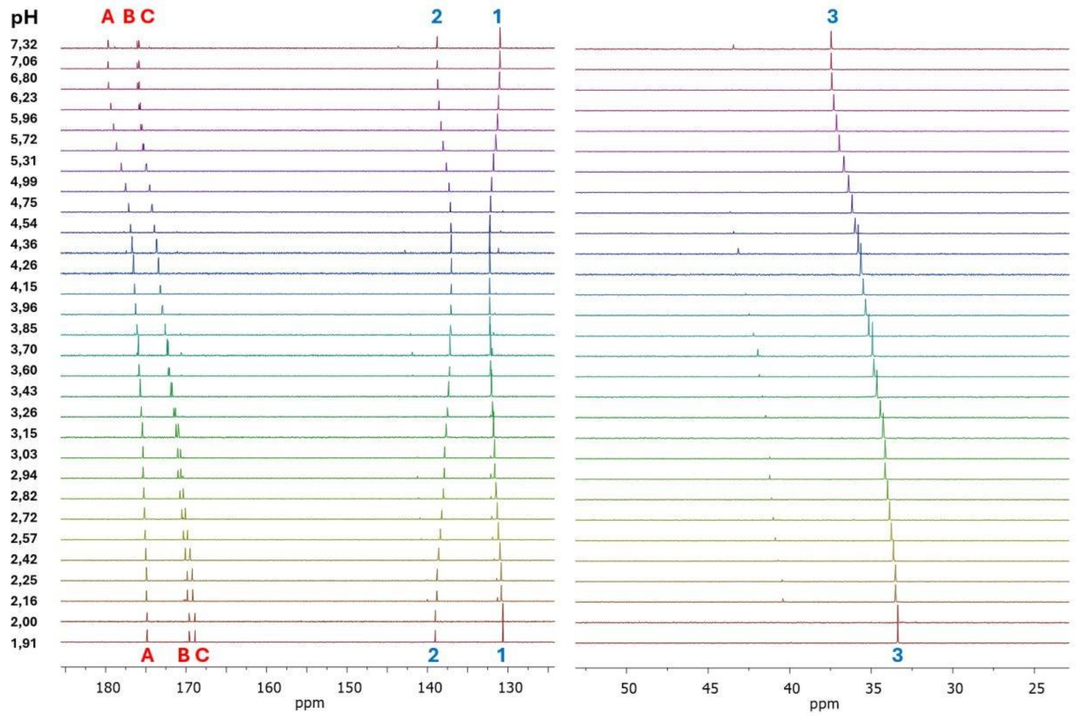

Figure S3. ^{13}C NMR spectra of *trans*-aconitic acid recorded in 9:1 (v/v) H_2O/D_2O mixture over the pH range 1.91-7.32 at c_L = 100 mmol dm^{-3}, $I = 0.15$ mol dm^{-3} in $KCl_{(aq)}$ and T = 298.15 K and

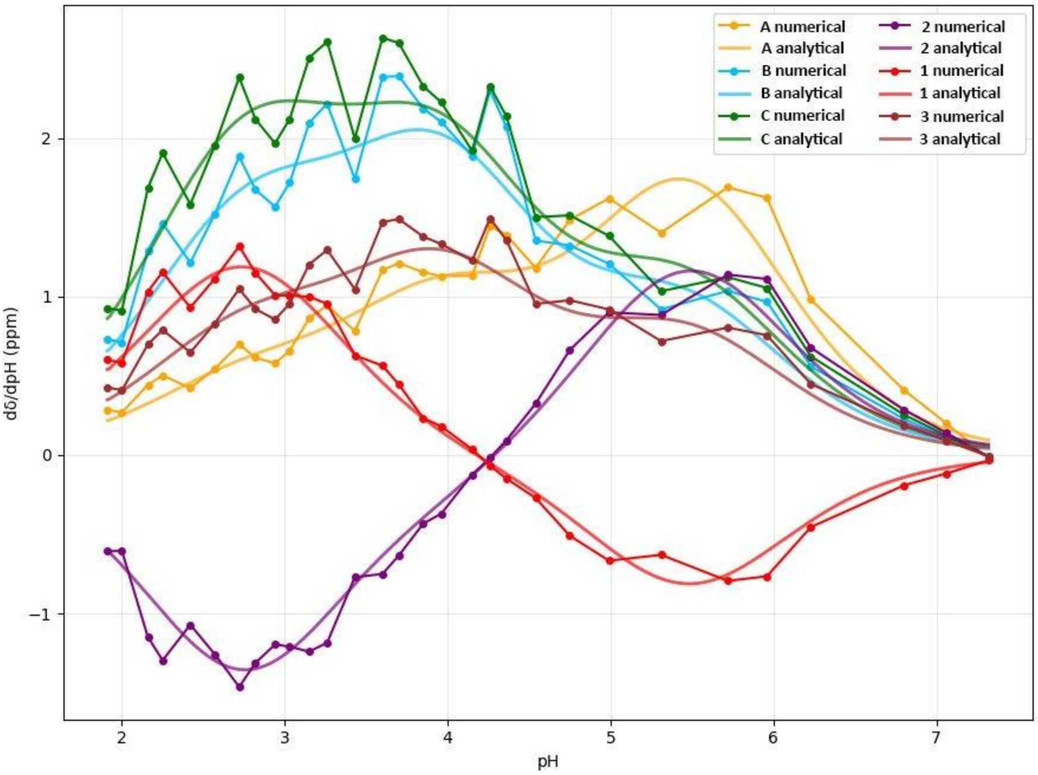

Figure S4. Comparison between numerical (symbols connected by lines) and analytical (solid lines) derivatives of the ^{13}C NMR chemical shifts with respect to pH, dδ/dpH, for all carbon atoms of *trans*-aconitic acid (A, B, C, 1, 2, and 3). Numerical derivatives were obtained from the experimental δ(pH) data, while analytical derivatives were calculated from a global triprotic Henderson–Hasselbalch model fitted simultaneously to all ^{13}C signals. The consistent reproduction of the experimental derivative profiles for both carboxyl and non-carboxyl carbons demonstrates that the extracted pK_a values describe global acid–base equilibria of the molecule. The correlated response of carbons 2 and 1 with carbons B and C, respectively, and of carbon 3 with carbon A, further supports the proposed deprotonation sequence and the propagation of electronic effects along the molecular framework.

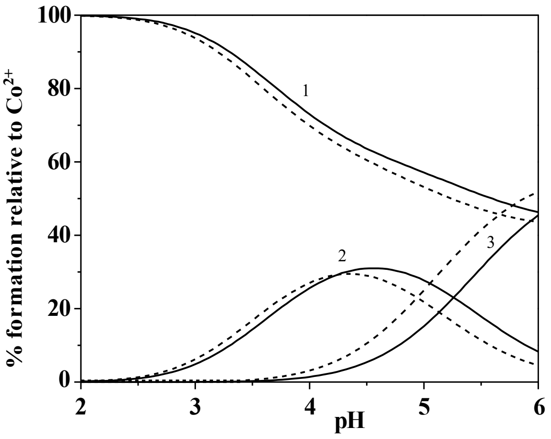

a)

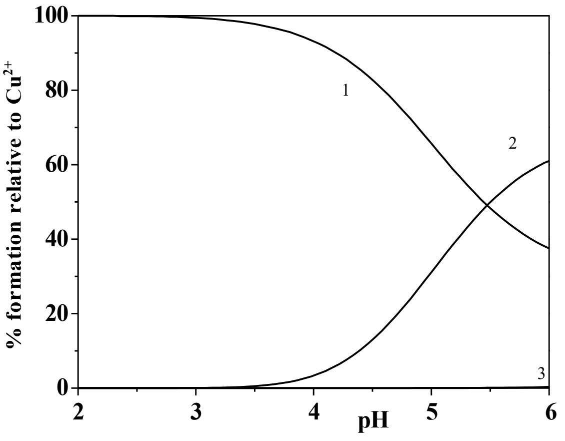

b)

Figure S5. Speciation diagrams of: a) Co^{2+}/L^{3-} species at $I = 0.15$ mol dm^{-3} (solid line) and $I = 1.00$ mol dm^{-3} (dashed line), $T = 298.15$ K. Species: 1 free Co^{2+}, 2 $CoHL^0$, 3 CoL^-; b) Cu^{2+}/L^{3-} complexes at $I = 0.15$ mol dm^{-3}, $T = 298.15$ K, species: 1 free Cu^{2+}, 2 CuL^-, 3 $Cu_2(OH)_2^{2+}$. Analytical concentrations: $c_M = 0.001$ mol dm^{-3}; $c_L = 0.003$ mol dm^{-3}.

a)

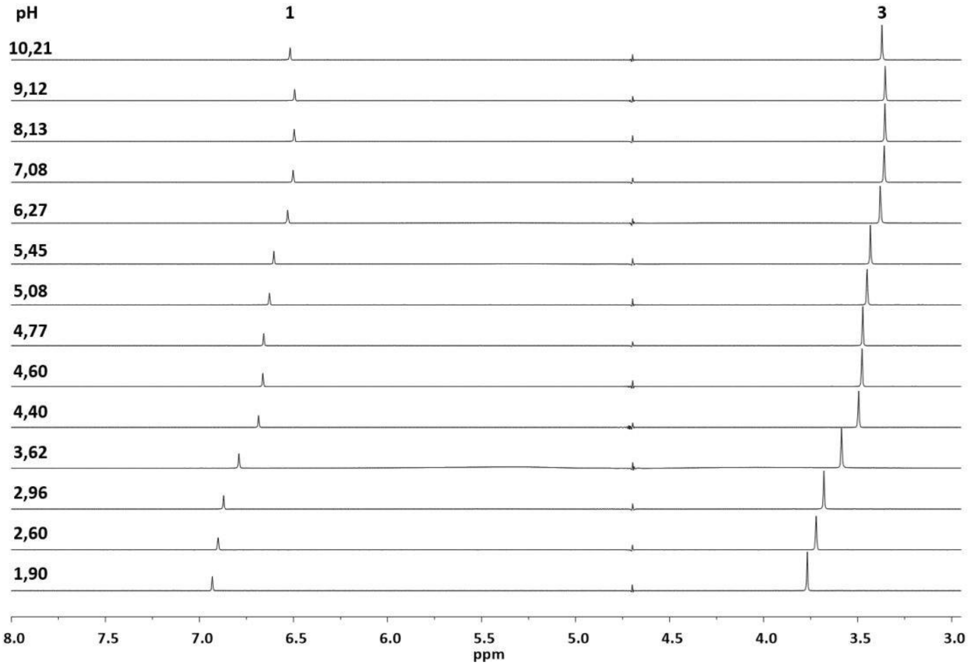

b)

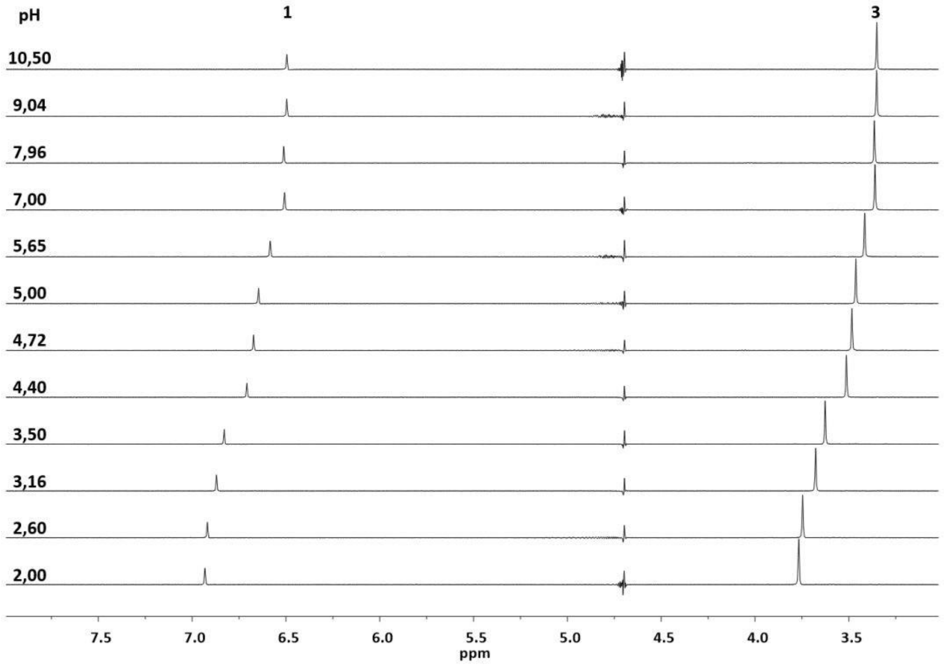

Figure S6. ^{1}H NMR spectra of Ca^{2+} complexes of *trans*-aconitic acid recorded in 9:1 (v/v) H_2O/D_2O mixture at I = 0.15 mol dm^{-3} in $KCl_{(aq)}$ and T = 298.15 K. a) c_{Ca} = 1.6 mmol dm^{-3}, c_L = 5.0 mmol dm^{-3}; b) c_{Ca} = 2.0 mmol dm^{-3}, c_L = 2.0 mmol dm^{-3}.

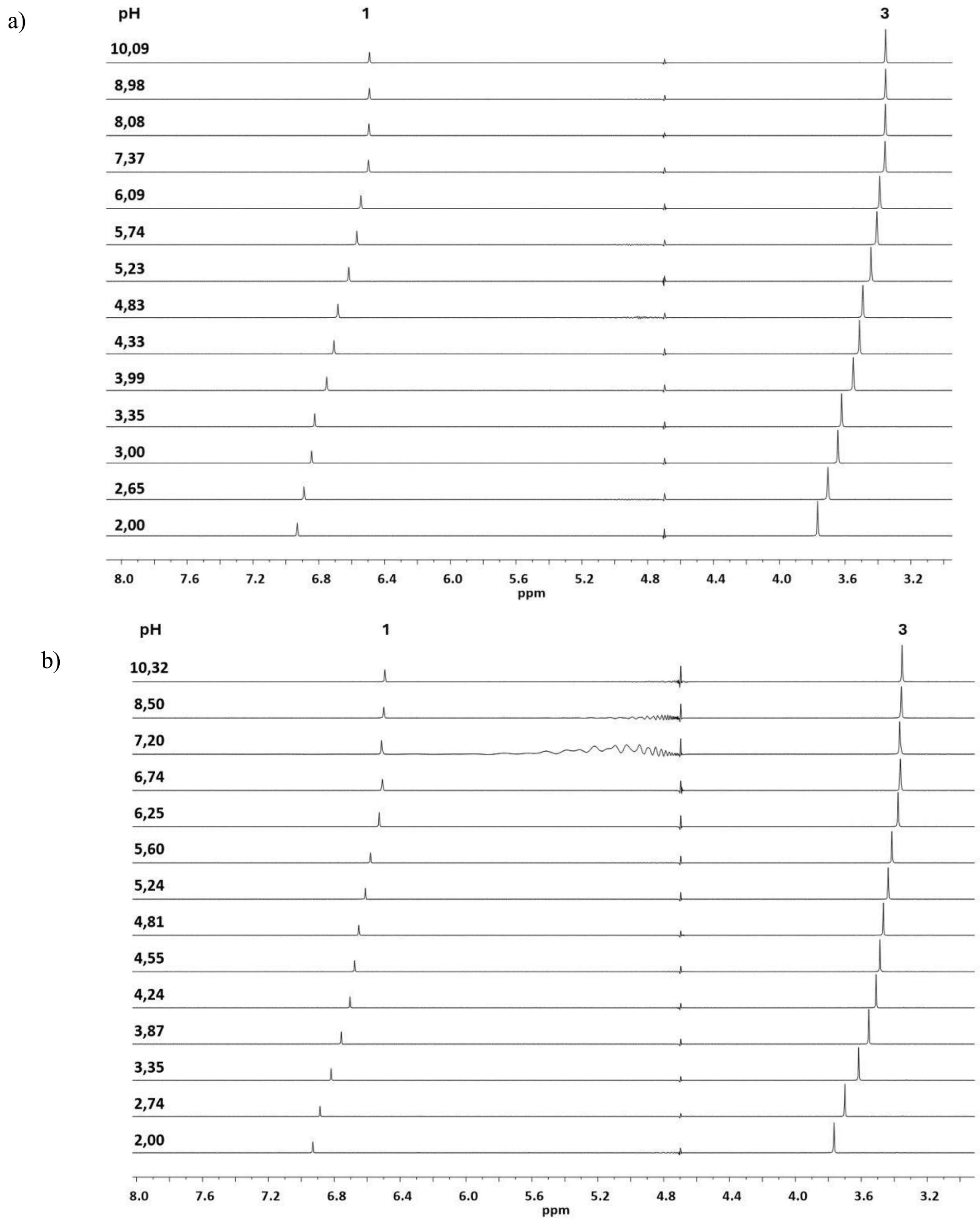

Figure S7. ^{1}H NMR spectra of Mg^{2+} complexes of *trans*-aconitic acid recorded in 9:1 (v/v) H_2O/D_2O mixture at I = 0.15 mol dm^{-3} in $KCl_{(aq)}$ and T = 298.15 K. a) c_{Mg} = 1.6 mmol dm^{-3}, c_L = 5.0 mmol dm^{-3}; b) c_{Mg} = 2.0 mmol dm^{-3}, c_L = 2.0 mmol dm^{-3}.

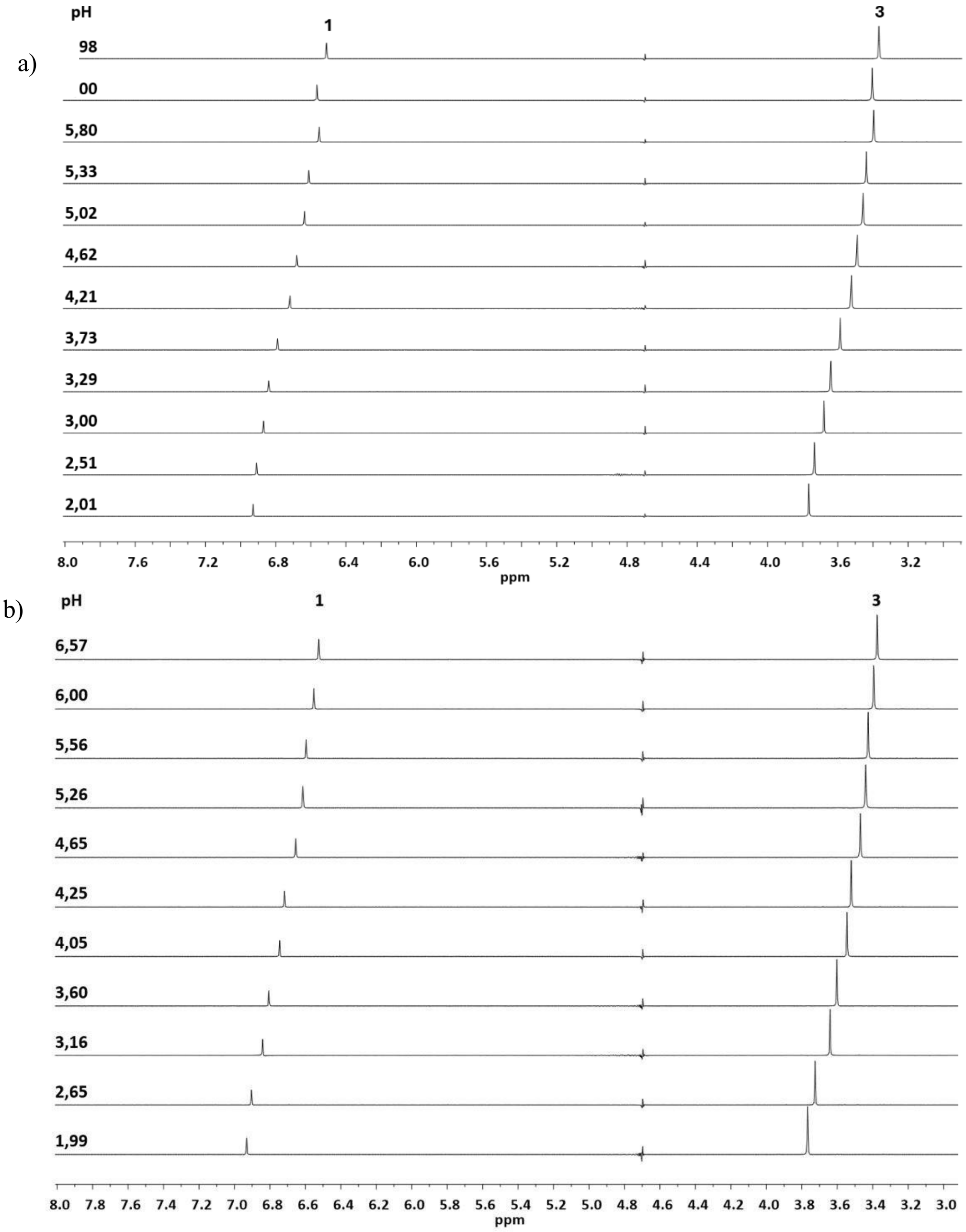

Figure S8. ^{1}H NMR spectra of Zn^{2+} complexes of *trans*-aconitic acid recorded in 9:1 (v/v) H_2O/D_2O mixture at $I = 0.15$ mol dm^{-3} in $KCl_{(aq)}$ and $T = 298.15$ K. a) $c_{Zn} = 1.6$ mmol dm^{-3}, $c_L = 5.0$ mmol dm^{-3}; b) $c_{Zn} = 2.0$ mmol dm^{-3}, $c_L = 2.0$ mmol dm^{-3}.

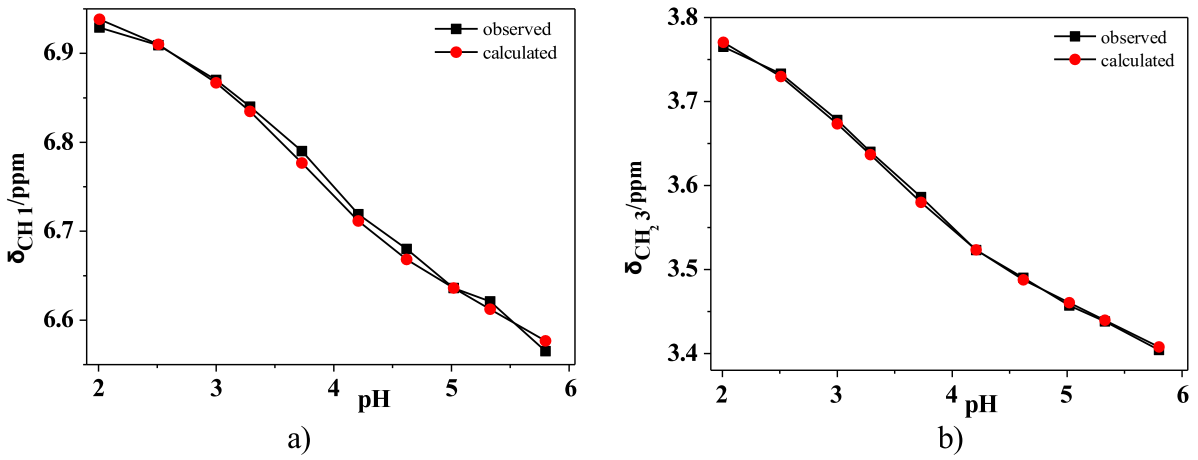

Figure S9. Observed (■) and calculated (•)proton chemical shifts values of a) nucleus 1 and b) nucleus 3 of *trans*-aconitic acid *vs* pH at c_{Zn} = 1.60 mmol dm^{-3}, c_L = 5.00 mmol dm^{-3}, I = 0.15 mol dm^{-3} in $KCl_{(aq)}$ and T = 298.15 K.

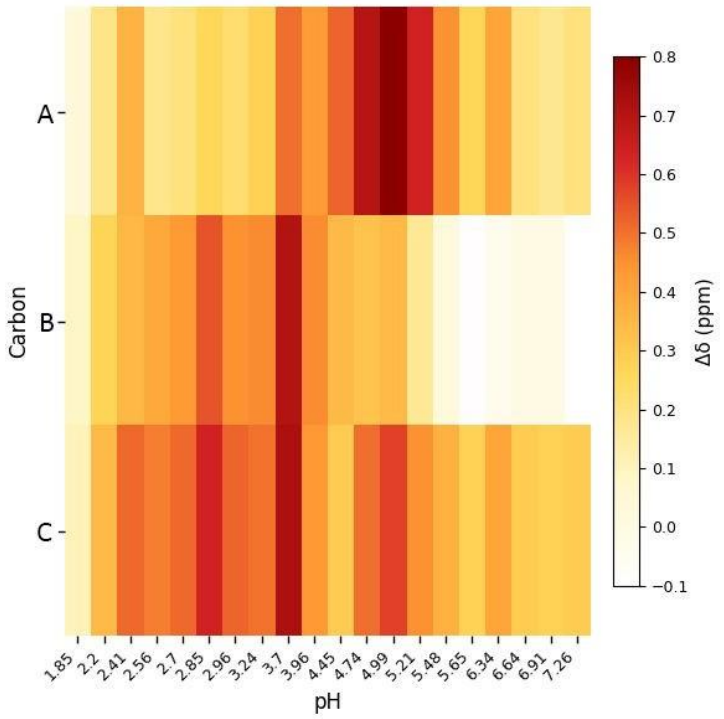

Figure S10. Heatmap representation of the chemical shift perturbations (Δδ = δ(*t*AA – Mg^{2+}) – δ(*t*AA)) for carbon atoms A, B, and C of *trans*-aconitic acid as a function of pH. Free *t*AA chemical shifts were obtained by cubic interpolation of the *t*AA titration curves. Positive Δδ values indicate Mg^{2+} induced deshielding. The heatmap highlights the pH-dependent magnitude of chemical shift perturbations for each site.

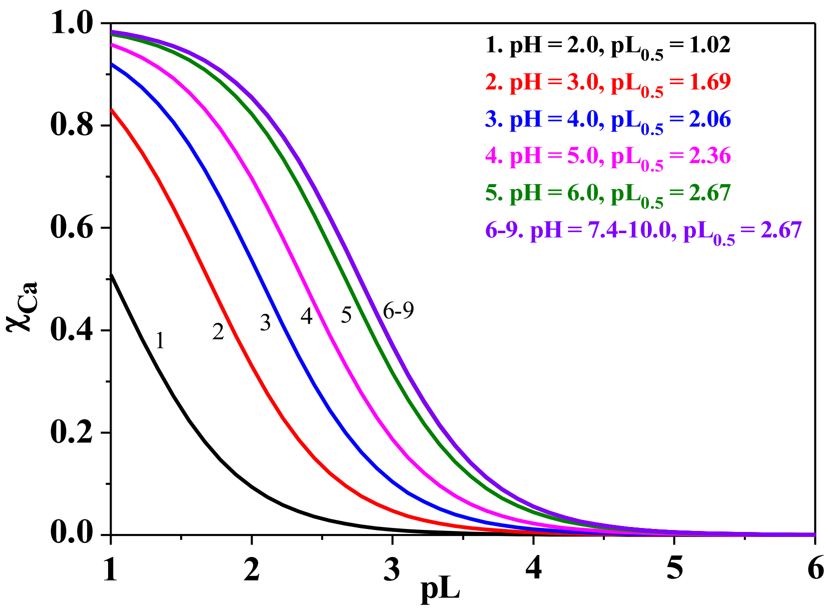

Figure S11. Sequestration diagrams of *trans*-aconitic acid towards Ca^{2+} at different pHs, I = 0.15 mol dm^{-3} in $KCl_{(aq)}$ and T = 298.15 K.

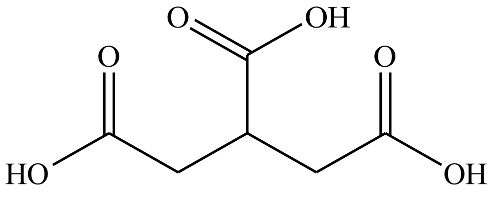

TriCarballylic Acid (TA)

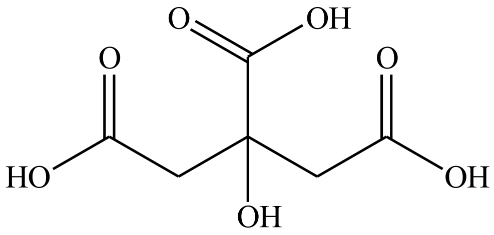

Citric Acid (CA)

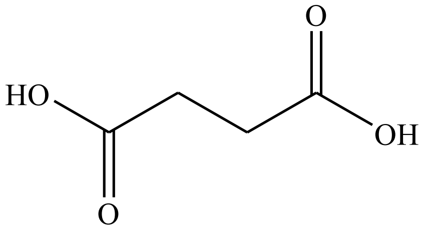

Succinic Acid (SA)

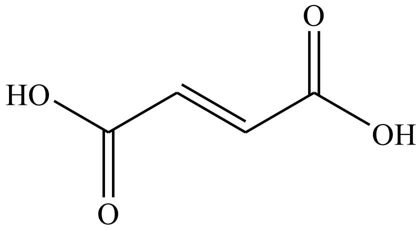

Fumaric Acid (FA)

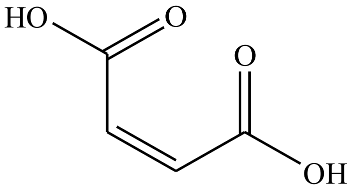

Maleic Acid (MA)

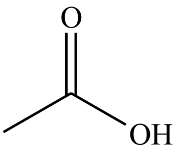

ACetic Acid (ACA)

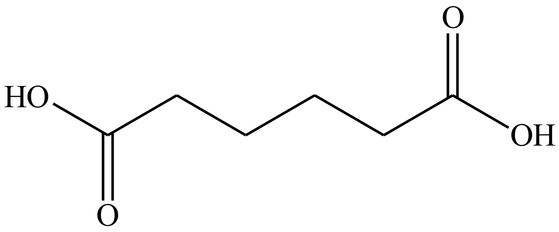

ADipic Acid (ADA)

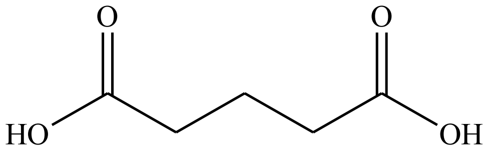

Glutaric Acid (GA)

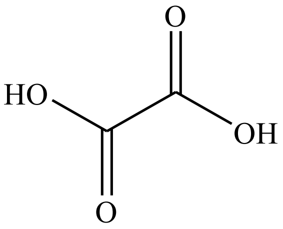

Oxalic Acid (OA)

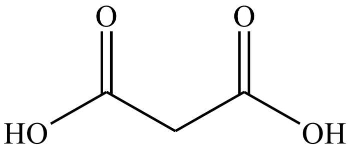

MAlonic Acid (MAA)

Figure S12. Structures of mono-, di- and tricarboxylic acids considered to perform literature comparisons with respect to *t*AA.

REFERENCES

1. C. De Stefano, P. Princi, C. Rigano and S. Sammartano, Computer analysis of equilibrium data in solution. ESAB2M: an improved version of the ESAB program, *Annali di Chimica*, 1987, **77**, 643-675.
2. C. De Stefano, P. Mineo, C. Rigano and S. Sammartano, Ionic Strength Dependence of Formation Constants. XVII. The Calculation of Equilibrium concentrations and Formation Constants., *Ann. Chim. (Rome)*, 1993, **83**, 243-277.
3. P. Gans, A. Sabatini and A. Vacca, Investigation of equilibria in solution. Determination of equilibrium constants with the HYPERQUAD suite of programs, *Talanta*, 1996, **43**, 1739-1753.
4. C. De Stefano, S. Sammartano, P. Mineo and C. Rigano, in *Marine chemistry-an environmental analytical chemistry approach*, Kluwer Academic Publishers, 1997, pp. 71-83.
5. C. Frassineti, S. Ghelli, P. Gans, A. Sabatini, M. S. Moruzzi and A. Vacca, Nuclear Magnetic Resonance as a Tool for Determining Protonation Constants of Natural Polyprotic Bases in Solution, *Anal. Biochem.*, 1995, **231**, 374-382.
6. S. J. Markich and P. L. Brown, *Thermochemical data for environmentally-relevant elements*, Report 1030-7746 0 642 59972 6, Australia, 1999.
7. E. Campi, G. Ostacoli, M. Meirone and G. Saini, Stability of the complexes of tricarballylic and citric acids with bivalent metal ions in aqueous solution, *J. Inorg. Nucl. Chem.*, 1964, **26**, 553-564.
8. E. É. Kiss, M. Jezowska-Bojczuk and T. Kiss, Complexes of aminoohosphonates part 9.1 Copper (II) complexes of citric derivatives, *J. Coord. Chem.*, 1996, **40**, 157-166.
9. P. M. May, D. Rowland, E. Königsberger and G. Hefter, JESS, a Joint Expert Speciation System--IV: a large database of aqueous solution physicochemical properties with an automatic means of achieving thermodynamic consistency, *Talanta*, 2010, **81**, 142-148.
10. R. J. Motekaitis and A. E. Martell, Potentiometry of mixtures: metal chelate stability constants of 1-hydroxy-3-oxapentane-1,2,4,5-tetracarboxylic acid and 3,6-dioxaoctane-1,2,4,5,7,8-hexacarboxylic acid, *Inorganic Chemistry*, 1989, **28**, 3499-3503.
11. L. D. Pettit and K. Powell, The IUPAC stability constants database, *Chem. Int*, 2006, **56**, 14-15.
